# Characterization of the molecular mechanisms that govern anti-Müllerian hormone synthesis and activity

**DOI:** 10.1096/fj.202301335RR

**Published:** 2024-01

**Authors:** William A. Stocker, James A. Howard, Shreya Maskey, Haitong Luan, Sophie G. Harrison, Kaitlin N. Hart, Lucija Hok, Thomas B. Thompson, Kelly L. Walton, Craig A. Harrison

**Affiliations:** 1 Department of Physiology, Monash Biomedicine Discovery Institute, Monash University, Clayton, Victoria, Australia; 2 Department of Pharmacology and Systems Physiology, University of Cincinnati, Cincinnati, Ohio, USA; 3 Department of Molecular and Cellular Biosciences, University of Cincinnati, Cincinnati, Ohio, USA; 4 School of Biomedical Sciences, The University of Queensland, Brisbane, Queensland, Australia

**Keywords:** AMH, anti-Müllerian hormone, folliculogenesis, ovary, protein engineering, TGF-β superfamily

## Abstract

The roles of anti-Müllerian hormone (AMH) continue to expand, from its discovery as a critical factor in sex determination, through its identification as a regulator of ovarian folliculogenesis, its use in fertility clinics as a measure of ovarian reserve, and its emerging role in hypothalamic–pituitary function. In light of these actions, AMH is considered an attractive therapeutic target to address diverse reproductive needs, including fertility preservation. Here, we set out to characterize the molecular mechanisms that govern AMH synthesis and activity. First, we enhanced the processing of the AMH precursor to >90% by introducing more efficient proprotein convertase cleavage sites (RKKR or ISSRKKRSVSS [SCUT]). Importantly, enhanced processing corresponded with a dramatic increase in secreted AMH activity. Next, based on species differences across the AMH type II receptor-binding interface, we generated a series of human AMH variants and assessed bioactivity. AMH_SCUT_ potency (EC_50_ 4 ng/mL) was increased 5- or 10-fold by incorporating Gln^484^Met/Leu^535^Thr (EC_50_ 0.8 ng/mL) or Gln^484^Met/Gly^533^Ser (EC_50_ 0.4 ng/mL) mutations, respectively. Furthermore, the Gln^484^Met/Leu^535^Thr double mutant displayed enhanced efficacy, relative to AMH_SCUT_. Finally, we identified residues within the wrist pre-helix of AMH (Trp^494^, Gln^496^, Ser^497^, and Asp^498^) that likely mediate type I receptor binding. Mutagenesis of these residues generated gain- (Trp^494^Phe or Gln^496^Leu) or loss- (Ser^497^Ala) of function AMH variants. Surprisingly, combining activating type I and type II receptor mutations only led to modest additive increases in AMH potency/efficacy. Our study is the first to characterize AMH residues involved in type I receptor binding and suggests a step-wise receptor-complex assembly mechanism, in which enhancement in the affinity of the ligand for either receptor can increase AMH activity beyond the natural level.

## 1 | INTRODUCTION

Anti-Müllerian hormone (AMH), a member of the transforming growth factor-β (TGF-β) superfamily, was first identified in the 1940s as a critical factor in male sex differentiation,^1^ via its ability to drive regression of Müllerian ducts.^2^ In humans, mutations in AMH or its type II receptor (AMHR2) lead to persistent Müllerian duct syndrome (PMDS), an autosomal recessive disorder characterized by the persistence of Müllerian derivatives (uterus, fallopian tubes, and upper portion of the vagina) in otherwise normally virilised males.^3^ In the female human fetus, AMH is not expressed until ~36–38 weeks gestation^4^ (in female mice, *Amh* is first expressed at post-natal Day 6^5^), at which point the Müllerian ducts have already developed into the fallopian tubes and uterus. However, from around the time of birth in all female mammals, AMH is secreted by granulosa cells of small growing ovarian follicles (e.g., secondary and early antral follicles).^6^ Studies on *Amh* knockout mice^7^ and *ex vivo* ovarian culture systems in various species,^8^ identified a role for AMH in blocking primordial follicle activation. In the absence of *Amh* in mice, primordial follicles were recruited at an accelerated rate and resulted in depletion of the ovarian reserve at a younger age.^7^ Intriguingly, AMH functions as a survival factor that stimulates growth and antrum formation during the culture of pre-antral follicles from non-human primates.^9,10^ AMH also reduces the sensitivity of follicles to FSH across species, indicating that it is only when AMH expression declines that a follicle can undergo FSH-dependent cyclic recruitment, and proceed towards ovulation.^9,11–13^ Serum AMH levels in females peak at ~25 years and then decline steadily to undetectable levels at the time of menopause.^11^ As such, serum AMH is a reliable measure of the “ovarian reserve,” that is the quantity and quality of primordial follicles.^8^ Clinically, AMH assays are used to assess the ovarian reserve during fertility treatments, and after gonadotoxic cancer treatments, or ovarian surgery.^14,15^ Thus, AMH not only acts to limit primordial follicle activation, but is also an important diagnostic marker for ovarian aging.

AMH, like other TGF-β family proteins, is synthesized as a precursor molecule with the N-terminal prodomain mediating folding and dimerization of the C-terminal mature domain.^16^ Dimeric precursors are cleaved by proprotein convertases^17^ and AMH is secreted from the cell non-covalently associated with its prodomain.^18^ Although precursor processing is essential for AMH bioactivity,^19,20^ the proportion of secreted AMH having undergone cleavage is typically only ~10%.^16,17^ Extracellularly, the prodomain likely localizes AMH in the vicinity of target cells via interactions with the extracellular matrix.^21,22^ AMH is displaced from its prodomain by its unique type II receptor, AMHR2.^18^ In a recent study, Hart *et al*.^23^ used x-ray crystallography to demonstrate that AMH engages AMHR2 using an extensive interface distinct from other type II receptors of the TGF-β family. Formation of the AMH/AMHR2 complex leads to recruitment and activation of the type I receptors, ALK2, ALK3, or ALK6, which potentiate downstream signaling via SMAD1/5/9 transcription factors.^24,25^ Recently, Meinsohn et al.^26^ used single-cell sequencing to demonstrate that AMH activation of this pathway suppressed neonatal follicle development by inducing a suppressive transcriptional program in granulosa cells.

The ability of AMH to limit primordial follicle recruitment makes it an attractive agent for slowing oocyte loss. Accordingly, it has been shown that supraphysiological doses of AMH can arrest folliculogenesis at the primary stage in mice.^27^ Indeed, AMH administration to mice resulted in reduced ovarian size, owing to the absence of growing follicles, but did not disrupt the ovarian reserve.^27^ Critically, AMH-mediated inhibition of primordial follicle recruitment was reversible, as discontinuation of AMH treatment restored folliculogenesis and fertility.^27^ In addition, AMH may represent a novel fertility preservation agent during chemotherapy. Chemotherapeutic agents damage the ovary by inducing apoptosis in growing follicles and upregulating Akt-dependent primordial follicle recruitment. Together, these processes lead to rapid “burnout” of the ovarian reserve.^28^ However, co-delivery of AMH with chemotherapeutics protected the ovarian reserve in mice.^27,29,30^ Together, these studies identified AMH as an attractive contraceptive/fertility-preservation agent.

In this study, we aimed to characterize the molecular mechanisms that govern AMH synthesis and activity. We first identified the regions of the prodomain important for AMH biosynthesis and further enhanced mature AMH production. Using the AMH/AMHR2 structure as a guide, we then generated several AMH gain-of-function variants. Finally, we identified AMH residues involved in type I receptor-binding and demonstrated that modification of these residues could also substantially enhance AMH activity. Together, our protein engineering has generated a series of AMH analogs with enhanced processing and activity, which has advanced our knowledge about how AMH forms a functional signaling complex with its type I and type II receptors.

## 2 | MATERIALS AND METHODS

### 2.1 | Generation of AMH mutant expression vectors

A mammalian expression vector (pcDNA3.1) containing a modified form of full-length human AMH cDNA (modifications included a 6×His epitope-tag immediately after Glu^29^, and a Gln^450^Arg substitution to enhance precursor cleavage) was kindly gifted by Dr Axel Themmen (Erasmus University, The Netherlands).^31^ We subsequently modified the cleavage site to the theoretically ideal “ISSRKKRSVSS” super-cut (SCUT) motif^32^ via overlap-extension PCR using custom-designed SCUT primers in combination with primers flanking the ORF (primer details provided in Table S1) and subcloned the modified cDNA into compatible sites of pCDNA3.1 (Thermo Fisher Scientific, Waltham, MA, USA). The AMH_SCUT_ construct was used as a template for further mutagenesis, undertaken with the QuikChange Lightning Site-Directed Mutagenesis Kit (Agilent Technologies, Santa Clara, CA, USA), with the exception of the ΔPro^30^-Pro^69^ and ΔPro^30^-Gly^119^ constructs, which were generated using the Q5^®^ Site-Directed Mutagenesis Kit (New England Biolabs, Ipswich, MA, USA).

### 2.2 | Transient expression of AMH variants in HEK293T cells

For the production of recombinant AMH variants, HEK293T cells were plated at 4 × 10^5^ cells/well in 12-well plates in Dulbecco’s Modified Eagle Medium (DMEM) supplemented with 10% fetal calf serum (FCS) and incubated at 37°C in 5% CO_2_. After overnight incubation, plasmid DNA (2.5 μg/well) was combined with polyethylenimine (PEI)-MAX (Polysciences, Warrington, PA, USA) and after 30 min DNA-PEI complexes were added directly to cells and incubated in OPTI-MEM (Life Technologies, Carlsbad, CA, USA) medium for 4 h, before replacing with fresh OPTI-MEM and incubating a further 90 h before collection of conditioned medium containing secreted AMH. To assess intracellular AMH, cells were lysed in 100 μL of 1% Triton X-100 while shaking on ice for 20 min.

### 2.3 | Western blotting to assess AMH protein production

Conditioned medium was concentrated 12.5-fold with Nanosep microconcentrators (3 kDa MW cut-off; Pall Life Sciences, Port Washington, NY, USA) before separation of reduced samples by 10% SDS-PAGE and Western blot. The primary antibody (mAb-5/6), targeted to a region near the C-terminus of AMH (used at a 1:5000 dilution), was from Abcam (Cambridge, UK). The secondary antibody (diluted 1:10 000) was horseradish peroxidase-conjugated anti-mouse IgG (GE Healthcare, Buckinghamshire, UK), with detection of immunoreactive proteins using Lumi-light chemiluminescence reagents (Roche, Basel, Switzerland) and a ChemiDoc^™^ MP system (Bio-Rad, Hercules, CA, USA) with Image Lab^™^ software (Bio-Rad). A horseradish peroxidase-conjugated anti-GAPDH monoclonal antibody (diluted 1:10 000) was used as an internal control (Cell Signaling Technology, Danvers, MA, USA). The AMH prodomain was detected with a mouse-derived monoclonal His-tag antibody (R&D Systems, Minneapolis, MN, USA; used at a 1:1000 dilution), targeted to the 6×His epitope-tag located at the N-terminus of the prodomain.

### 2.4 | Large-scale production and purification of AMH variants

For larger-scale production, HEK293T cells were plated at 8 × 10^6^ cells/plate in 15 cm plates in DMEM supplemented with 10% FCS and incubated at 37°C in 5% CO_2_. After overnight incubation, plasmid DNA (60 μg/plate) was combined with PEI-MAX, and after 30 min DNA-PEI complexes were added directly to cells and incubated in OPTI-MEM medium for 4 h, before replacing with fresh OPTI-MEM containing 0.02% bovine serum albumin (BSA) and incubating a further 90 h before collection. Conditioned medium (~200 mL) was concentrated (Centricon Plus-70, 3 kDa MW cut-off; Merck Millipore, Burlington, MA, USA) and resuspended in binding buffer (50 mM phosphate buffer, 300 mM NaCl, pH 7.4) to a final volume of 5 mL. The concentrated medium was subjected to purification by immobilized metal affinity chromatography (IMAC) using Ni-NTA agarose (Thermo Fisher Scientific). Bound proteins were eluted from the resin with elution buffer (50 mM phosphate buffer, 300 mM NaCl, 500 mM imidazole) and imidazole was removed from the preparation by dialysis against Dulbecco’s phosphate buffered saline (Life Technologies) using a Slide-A-Lyzer^®^ MINI Dialysis Device (2 mL 3.5 K MW cut-off; Thermo Fisher Scientific). Mass estimates for AMH were determined by Western blotting using recombinant human AMH mature protein (R&D Systems) as standards. Densitometry was assessed with Image Lab^™^ software (Bio-Rad).

### 2.5 | AMH-responsive HEK293T luciferase assays

HEK293T cells were plated at 1.5 × 10^4^ cells/well in 96-well plates in DMEM supplemented with 10% FCS and incubated at 37°C in 5% CO_2_. After overnight incubation, Lipofectamine 2000 (Life Technologies) was used according to the manufacturer’s instructions to transfect cells with 100 ng/well of plasmid DNA, consisting of 4xBRE-luc (98.9 ng), AMHR2 (0.8 ng) and ALK2 (0.3 ng), diluted in OPTI-MEM. Approximately 24 h after transfection, cells were treated with increasing doses of AMH variants diluted in serum-free medium (DMEM high glucose supplemented with 1 mM sodium pyruvate [Life Technologies] and 0.01% BSA [Sigma-Aldrich, St. Louis, MO, USA]) and incubated overnight (~18 h) at 37°C in 5% CO_2_. The medium was then removed and cells lysed in solubilization buffer (26 mM glycylglycine [pH 7.8], 16 mM MgSO_4_, 4 mM EGTA, 900 μM dithiothreitol, 1% Triton X-100). The lysate was transferred to a white 96-well plate (Corning^®^ Costar^®^, Corning, NY, USA) and luminescence was measured immediately after the addition of the substrate luciferin (Promega, Madison, WI, USA), using a CLARIOstar microplate reader (BMG Labtech, Ortenberg, Germany).^23^

### 2.6 | AMH-responsive COV434 luciferase assays

COV434 cells (originally classified as a human granulosa tumor cell line^33,34^; although this has recently been contested, and are now suggested to be a small cell carcinoma of the ovary, hypercalcemic type^35^) were plated (7.5 × 10^4^ cells/well) in DMEM with 10% FCS into 48-well plates and incubated at 37°C in 5% CO_2_. The following day, Lipofectamine 3000 (Life Technologies) was used according to the manufacturer’s instructions to transfect cells with 250 ng/well of plasmid DNA, consisting of: 4xBRE-luc (248.4 ng) and AMHR2 (1.6 ng), diluted in OPTI-MEM. Approximately 24 h after transfection, cells were treated with increasing doses of AMH variants diluted in low-serum medium (DMEM high glucose supplemented with 50 mM HEPES [Life Technologies] and 0.2% FCS) and incubated overnight (~18 h) at 37°C in 5% CO_2_. The medium was then removed and cells lysed in solubilization buffer (26 mM glycylglycine [pH 7.8], 16 mM MgSO_4_, 4 mM EGTA, 900 μM dithiothreitol, 1% Triton X-100). The lysate was transferred to a white 96-well plate and luminescence was measured immediately after the addition of the substrate luciferin, using a CLARIOstar microplate reader.^36^

### 2.7 | Computational analysis of AMH variants

Alphafold 2.3.0 was used to generate models of mutant AMH constructs.^37,38^ Five AMBER relaxed models were generated for each mutant and the top-ranked model was used for the subsequent molecular dynamics (MD) simulations using the AMBER16 software suite.^39^ Input FASTAs for AMH and AMHR2 were truncated to the amino acid coverage of Protein Data Bank (PDB) ID 7L0J, while ALK2 input FASTAs were aligned and truncated to the amino acid coverage of ALK3 within PDB ID 2GOO. The constructed complexes of wild-type and mutant AMH dimers with the corresponding type I or type II receptor were solvated in a truncated octahedral box of TIP3P water molecules spanning an 8 Å-thick buffer. All systems were parametrized with the ff14SB force field and submitted to geometry optimization employing periodic boundary conditions in all directions. The optimized systems were gradually heated from 0 to 300 K and equilibrated at 30 ps using *NVT* conditions, followed by productive MD simulations of 150 ns utilizing a time step of 2 fs at constant pressure (1 atm) and temperature (300 K). The Particle Mesh Ewald method was used for efficient long-range electrostatic calculations, while non-bonded interactions were truncated at 11.0 Å. The equilibrated part of trajectories was subjected to the molecular mechanics generalized Born surface area (MM-GBSA) analysis to estimate ligand-receptor affinities.^40^ Calculated binding free energies (Δ*G*_BIND_) were decomposed into individual amino acid contributions on a *per-residue* basis.

### 2.8 | Statistical analysis

Differences in mature AMH secretion were assessed with ordinary one-way analysis of variance (ANOVA) and then Dunnett’s multiple comparisons test, except for the comparison between AMH_SCUT_ and the Ala^515^Val variant, which was assessed with an unpaired *t*-test. Differences in AMH cleavage efficiency were assessed with ordinary one-way ANOVA then Tukey’s multiple comparisons test. All statistical analyses were performed using GraphPad Prism 9.0. Data are presented as the mean ± SD. Significant differences were defined as: *p* < .05 (*), *p* < .01 (**), *p* < .001 (***), or *p* < .0001 (****).

## 3 | RESULTS

### 3.1 | Residues at the N-terminus of the AMH prodomain direct folding and secretion of the mature protein

**FIGURE 1:**
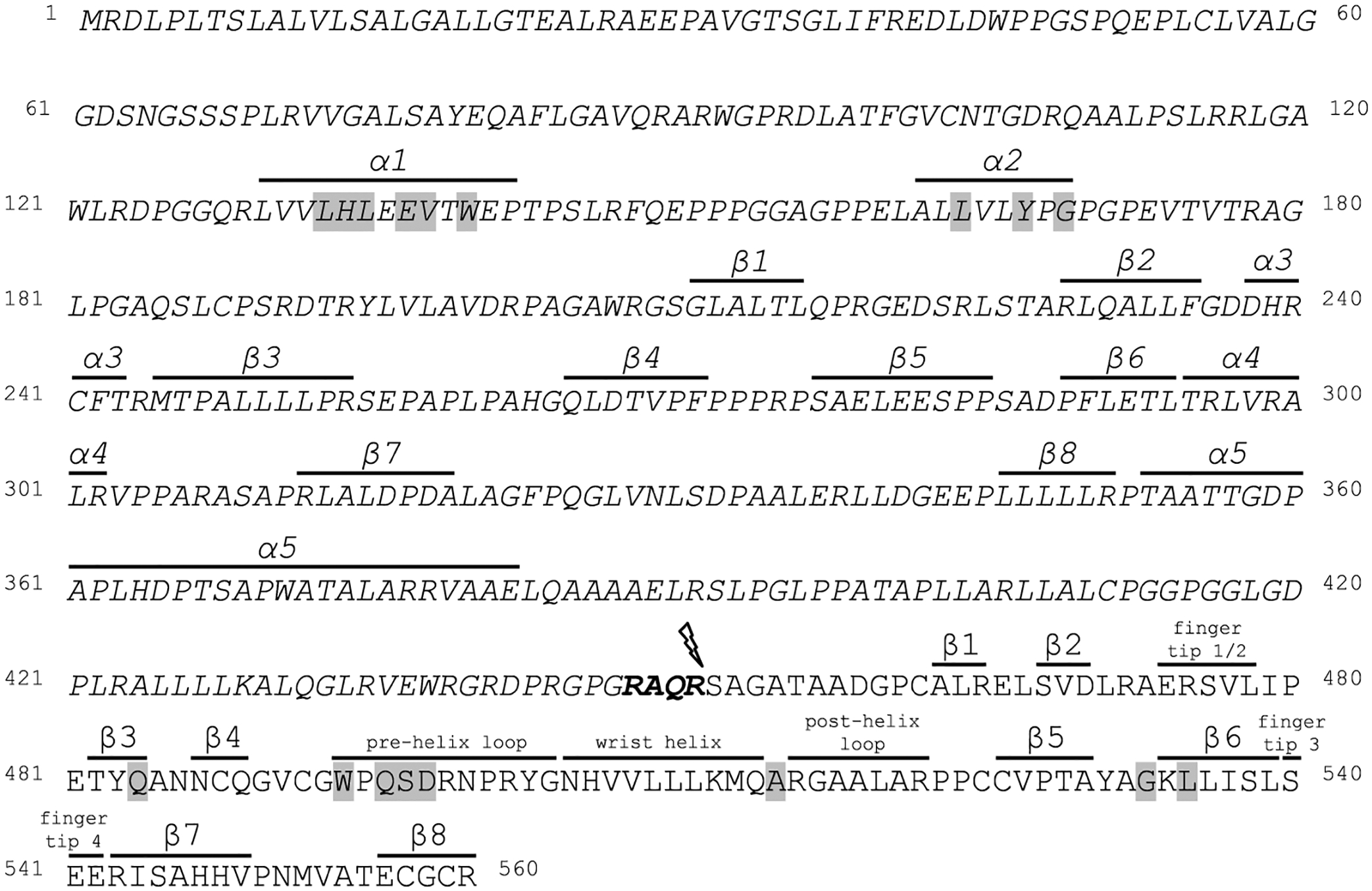
Human AMH amino acid sequence. Sequence of human AMH, with the prodomain indicated by italics. The residues are numbered according to the first residue of the signal peptide. The cleavage recognition motif RAQR, at the end of the prodomain, is in bold and the cleavage site marked (lightning bolt). Secondary structure elements (α-helices and β-sheets) are depicted above the sequence, with prodomain elements based on Hinck *et al*.^44^ and mature domain elements from Hart *et al*.^23^ Residues modified by *in vitro* site-directed mutagenesis are shaded. AMH, anti-Müllerian hormone.

Our aim in this study was to characterize the regions of AMH critical for synthesis, cleavage, and binding to type I and type II receptors. Based on our understanding of these processes in other TGF-β proteins,^36,41–43^ we used *in vitro* mutagenesis to delete or modify specific residues within human AMH (Figure 1) and assessed the effects of these mutations on biological function.

**FIGURE 2:**
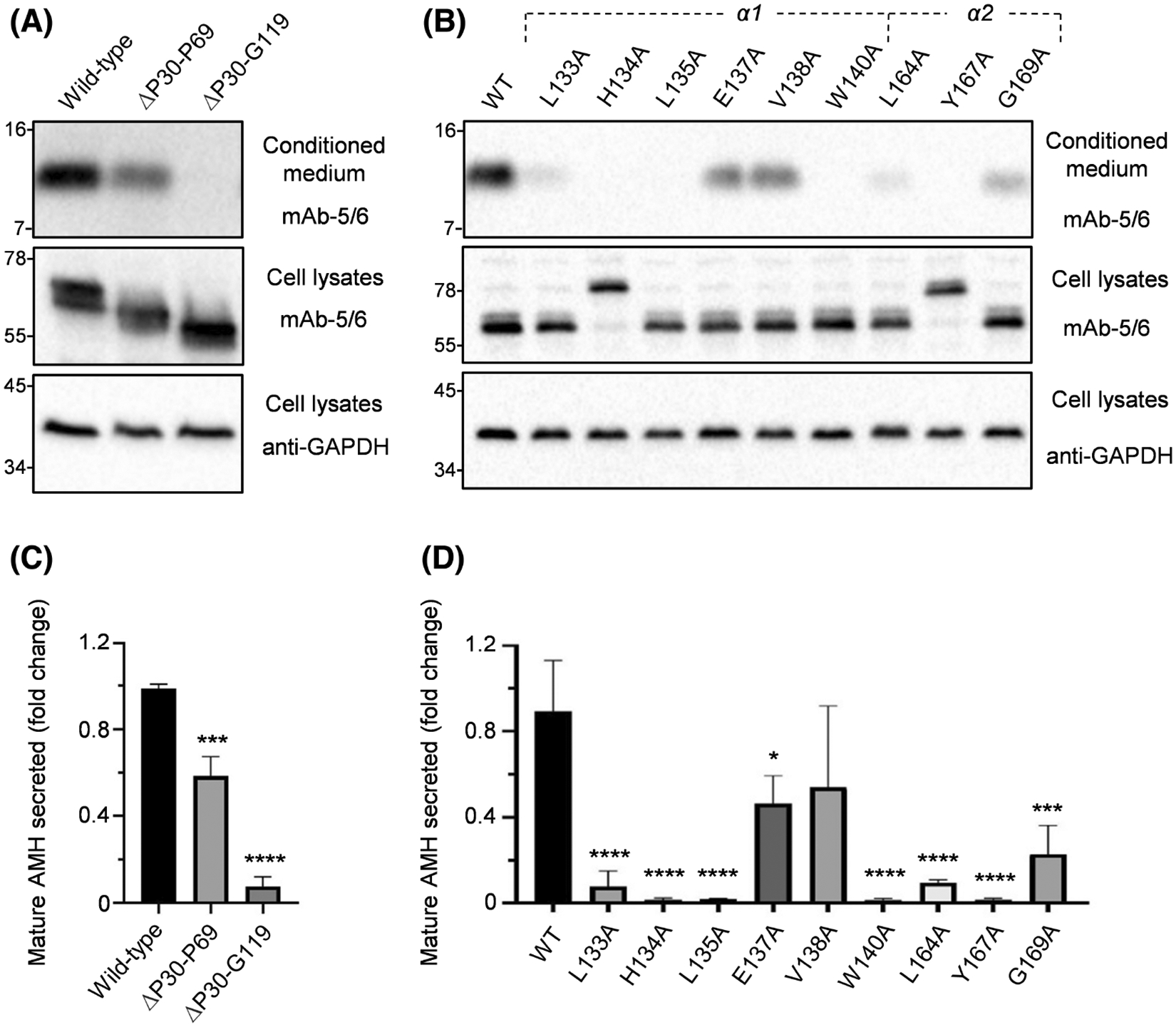
AMH prodomain regions required for secretion of mature protein. Portions of the AMH prodomain were removed (A) or mutated using *in vitro* mutagenesis (B). To determine the effects on AMH biosynthesis, conditioned medium and cell lysates from HEK293T cells transfected with either wild-type or mutant constructs were analyzed by Western blotting, with samples run under reducing conditions. Conditioned medium samples were probed with mAb-5/6, targeted to the AMH mature domain. Cell lysates were probed with mAb-5/6, or anti-GAPDH as a loading control. (C, D) Densitometric quantification of wild-type and mutant AMH mature domain secretion using the Bio-Rad ChemiDoc^™^ MP system and Image Lab^™^ software (Bio-Rad). Data are presented as the mean ± SD of three separate transfections, with representative Western blots shown in (A, B). Stars indicate significant differences at *p* < .05 (*), *p* < .001 (***), or *p* < .0001 (****) when compared to wild-type AMH. AMH, anti-Müllerian hormone.

The prodomain of AMH is 60–120 residues longer than most other TGF-β superfamily ligands, however, the function of this N-terminal extension is unclear.^44^ To assess whether these residues direct folding and secretion of the mature protein, we used *in vitro* mutagenesis to generate expression vectors in which 40 (Pro^30^-Pro^69^) or 90 (Pro^30^-Gly^119^) residues after the signal peptide were removed. Wild-type and mutant proteins were expressed in HEK293T cells and the conditioned medium and cell lysate were collected. Western blot analysis indicated that conditioned medium from cells transfected with wild-type AMH contained significant amounts of mature protein (12.5 kDa) (Figure 2A,C). In contrast, deletion of residues Pro^30^-Pro^69^ or Pro^30^-Gly^119^ decreased the quantity of mature protein secreted by 40% and 95%, respectively. The reduction in mature protein secreted was not due to less AMH precursor being translated, as analysis of cell lysates showed equal quantities of precursor (55–70 kDa, depending on the size of deletion) present intracellularly (Figure 2A,C).

The prodomain α1- and α2-helices of TGF-β family ligands direct the folding, dimerization, and secretion of mature proteins.^41,42,45^ Sequence alignment previously identified residues Leu^130^-Pro^142^ as the likely α1-helix in AMH, and residues Ala^162^-Gly^169^ as the likely α2-helix.^44^ Therefore, residues through these regions were substituted to alanine in order to assess their roles in mature protein secretion following expression in HEK293T cells. Wild-type mature protein was readily detectable in conditioned medium analyzed via Western blotting (Figure 2B,D). In contrast, mutation to alanine of residues Leu^133^, His^134^, Leu^135^, Trp^140^, Leu^164^, and Tyr^167^ led to a near complete loss of mature AMH secretion; while mutation of residues Glu^137^, Val^138^, and Gly^169^ to alanine had a less substantial impact. Analysis of cell lysates showed equal quantities of precursor present intracellularly, although the His^134^Ala and Tyr^167^Ala mutant precursors were larger (78 kDa) (Figure 2B,D). Together, these results indicate that the N-terminal extension and specific residues within the α1- and α2-helices of the prodomain mediate the correct folding and dimerization of AMH.

### 3.2 | Wild-type AMH is poorly cleaved, but cleavage efficiency and mature protein secretion can be enhanced with modifications to the processing site

**FIGURE 3:**
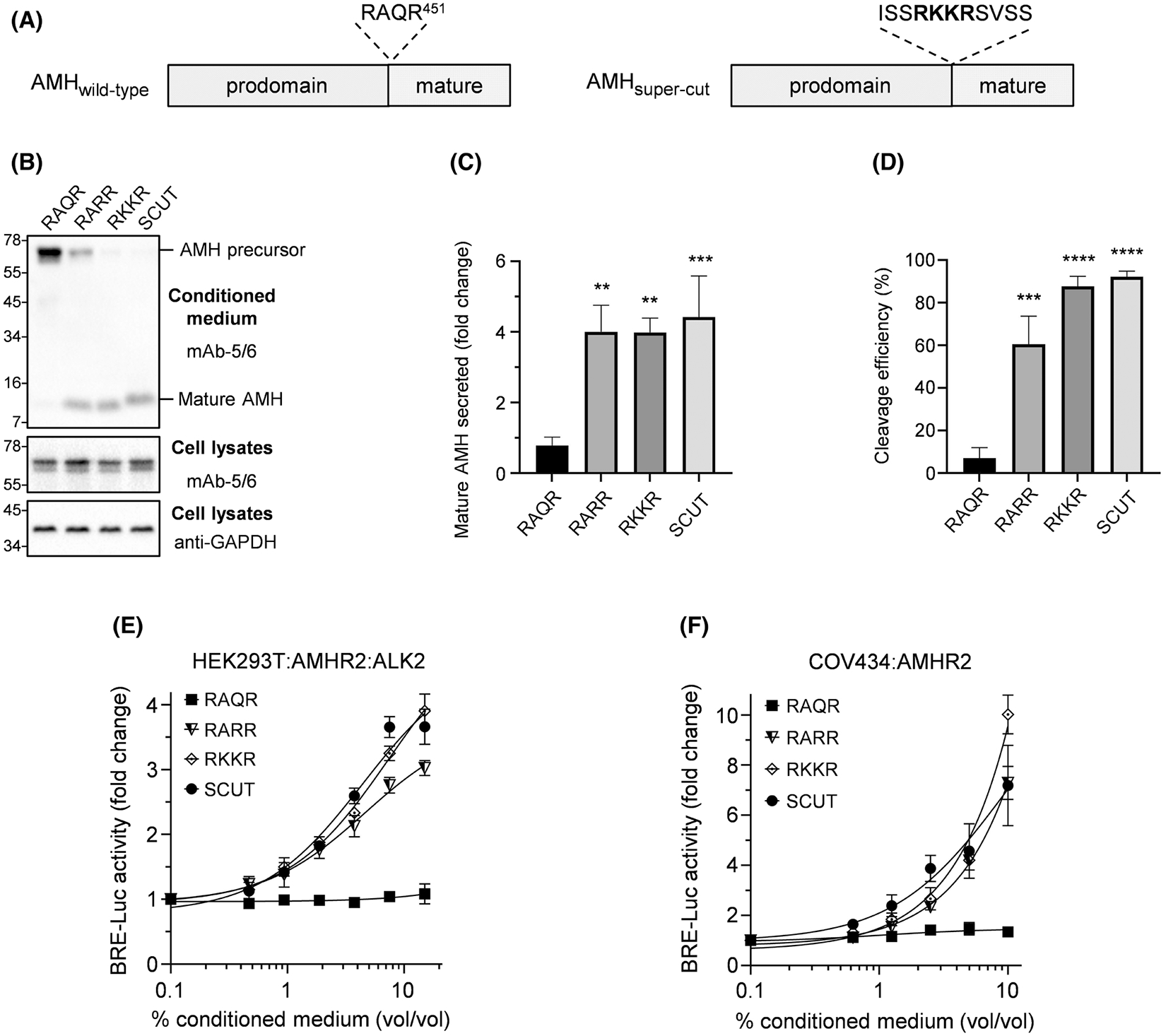
Modifications to improve cleavage of the AMH precursor. (A) The cleavage recognition motif (RAQR) in wild-type AMH was modified to the more efficient RKKR sequence, or the theoretically ideal super-cut sequence (ISSRKKRSVSS), using *in vitro* mutagenesis. (B) To determine the effects on AMH biosynthesis, conditioned medium and cell lysates from HEK293T cells transfected with either wild-type or mutant constructs were analyzed by Western blotting, with samples run under reducing conditions. Conditioned medium samples were probed with mAb-5/6, targeted to the AMH mature domain. Cell lysates were probed with mAb-5/6, or anti-GAPDH as a loading control. Densitometric quantification of wild-type and mutant AMH mature domain secretion (C) and cleavage efficiency (D) was performed using the Bio-Rad ChemiDoc^™^ MP system and Image Lab^™^ software (Bio-Rad). Data in (C, D) is presented as the mean ± SD of three separate transfections, with representative Western blots shown in (B). Stars indicate significant differences at *p* < .01 (**), *p* < .001 (***), or *p* < .0001 (****) when compared to wild-type AMH (RAQR). (E, F) SMAD1/5/9-responsive luciferase reporter (BRE-Luc) activity following treatment of cells with dilutions of conditioned medium collected from HEK293T cells transfected with wild-type or mutant AMH constructs. (E) HEK293T cells transfected with BRE-Luc, AMHR2, and ALK2. (F) COV434 cells transfected with BRE-Luc and AMHR2. Luciferase activity is presented as the mean ± SD of triplicates from representative experiments, relative to an adjusted value of 1.0 for the mean of the control wells. Experiments were repeated >3 times. AMH, anti-Müllerian hormone; AMHR2, AMH type II receptor.

To generate mature AMH, the precursor undergoes processing by kex2/subtilisin-like endoproteases after Arg^451^, which is the final residue in the cleavage recognition sequence “RAQR” (Figure 3A). However, cleavage of wild-type AMH is inefficient, with only ~10% of secreted AMH undergoing processing.^16,17^ While cleavage efficiency can be substantially improved via a Gln^450^Arg substitution, leading to a “RARR” cleavage recognition sequence, processing of the modified precursor is still not 100% efficient.^17,46^ Here, we determined whether the cleavage efficiency of AMH could be further enhanced by substituting the recognition sequence to “RKKR,” or by incorporating an ideal proprotein convertase recognition sequence “ISSRKKRSVSS” (termed SCUT).^32,43,47^

Following the expression of AMH variants with differing cleavage sites in HEK293T cells, the conditioned medium was analyzed by Western blot to assess the level of mature AMH secreted, and the efficiency of precursor cleavage (Figure 3B, top). While only 7% of the secreted wild-type AMH (RAQR) was processed, processing was enhanced with each cleavage site modification: RARR (60% processed), RKKR (90% processed), and SCUT (95% processed) (Figure 3D). Despite differences in cleavage efficiency, similar quantities of mature protein were secreted for all three cleavage site variants, a five-fold increase above wild-type AMH (Figure 3C). To confirm the AMH prodomain was also secreted following modification of the cleavage site, conditioned medium was probed with a monoclonal His-tag antibody, targeted to a 6×His epitope-tag located at the N-terminus of the prodomain. The prodomain was readily detectable for each cleavage variant, while wild-type AMH (RAQR) was detectable as an un-cleaved precursor (Figure S1).

To confirm that enhanced AMH processing corresponded with enhanced activity; HEK293T cells transfected with a SMAD1/5/9-responsive transcriptional reporter, together with the receptors AMHR2 and ALK2,^48^ were treated with equal dilutions of conditioned medium from cells expressing cleavage site variants (RAQR [*wild-type*], RARR, RKKR or SCUT) (Figure 3E). While medium containing wild-type AMH showed no activity at the doses tested, medium from cells expressing the cleavage site variants dose-dependently increased reporter activity. A similar result was also observed in COV434 cells transfected with a SMAD1/5/9-responsive transcriptional reporter and AMHR2 (Figure 3F). These findings indicate that enhanced AMH processing markedly increases AMH activity.

### 3.3 | Targeted mutation of the AMHR2-binding site identifies AMH variants with enhanced activity

**FIGURE 4:**
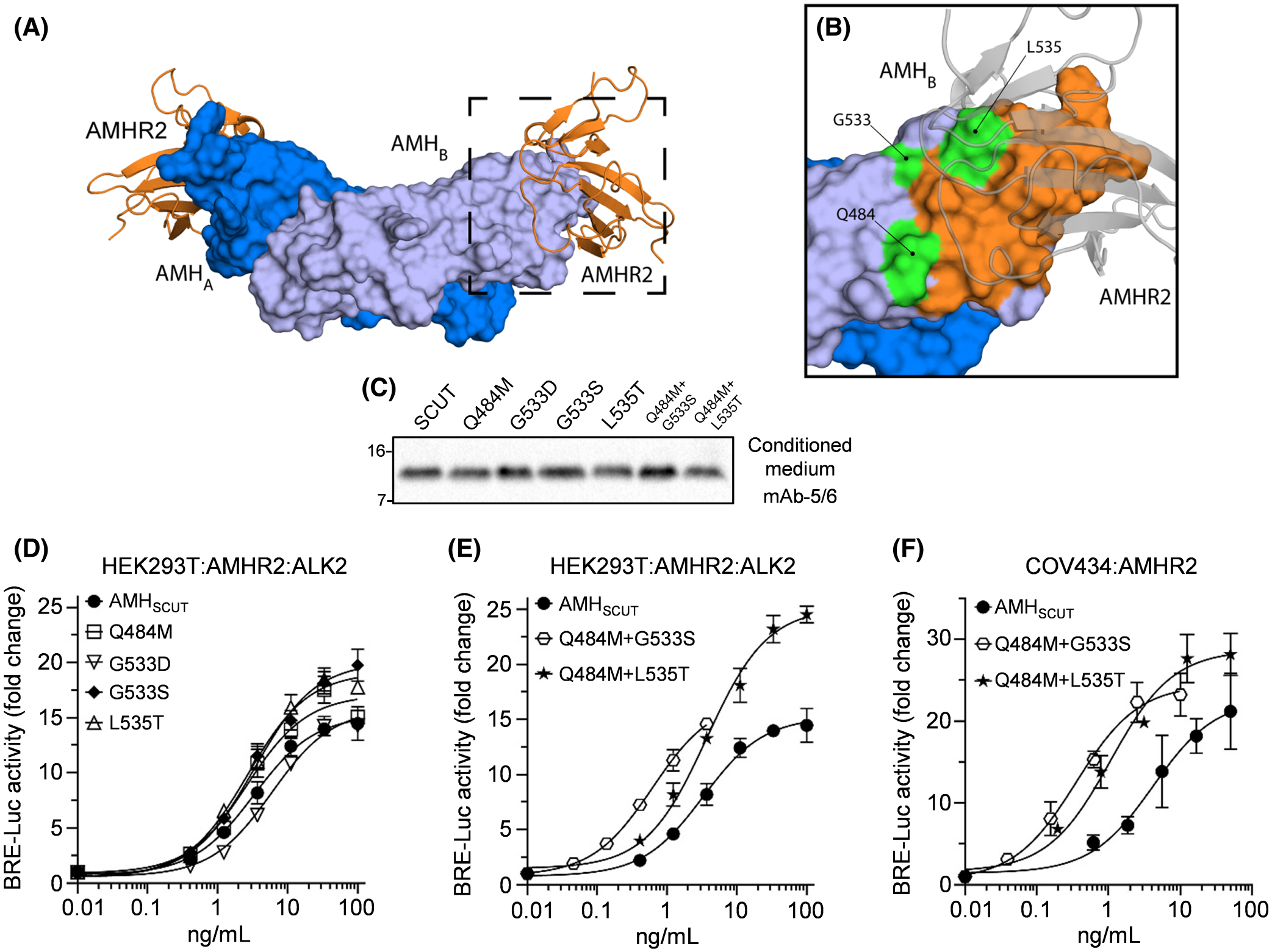
Modifications to the type II site. (A) Structure of dimeric human mature AMH bound at each knuckle by a single AMHR2 (PDB ID 7L0J). Each monomer of the AMH homodimer is colored in a different shade of blue. Each AMHR2 is colored orange. (B) Close-up view of the AMHR2-binding site of one AMH monomer. Atoms of AMH within 5 Å of AMHR2 are colored orange, while residues targeted by mutagenesis are colored green. (C) Residues in the AMHR2-binding epitope of AMH were mutated in the AMH_SCUT_ construct using *in vitro* mutagenesis. Conditioned medium from HEK293T cells transfected with AMH_SCUT_ or type II mutant constructs was analyzed by Western blotting using mAb-5/6, targeted to the AMH mature domain, with samples run under reducing conditions. (D–F) Dose–response curves of SMAD1/5/9-responsive luciferase reporter (BRE-Luc) activity following treatment of cells with IMAC purified AMH_SCUT_ or type II mutants. (D, E) HEK293T cells transfected with BRE-Luc, AMHR2, and ALK2. (F) COV434 cells transfected with BRE-Luc and AMHR2. Luciferase activity is presented as the mean ± SD of triplicates from representative experiments, relative to an adjusted value of 1.0 for the mean of the control wells. Experiments were repeated >3 times. AMH, anti-Müllerian hormone; AMHR2, AMH type II receptor; IMAC, immobilized metal affinity chromatography.

Dimeric mature AMH has the typical butterfly-shaped architecture of TGF-β superfamily ligands (Figure 4A) and is bound at each knuckle epitope by a separate AMHR2 receptor.^23^ Mutation of the ligand-side of the AMH/AMHR2 binding interface has previously identified several AMH residues (e.g., Lys^534^ and Ala^546^) that are essential for activity^23^; however, no AMH variants with enhanced activity have been reported. We compared the amino acid sequence of the human AMH mature domain with nine other species (Figure S2), with a particular focus on residues making direct contact with AMHR2, or within the vicinity of Lys^534^, which has a critical role in binding AMHR2.^23^

Intriguingly, two residues in close proximity to Lys^534^ in human AMH (Gln^484^ and Leu^535^) are distinct in koala AMH (Met^565^ and Thr^616^), while Gly^533^ in human AMH is either a serine or aspartic acid in birds and reptiles. Thus, we generated point- or compound mutants incorporating Gln^484^Met, Gly^533^Asp, Gly^533^Ser, and/or Leu^535^Thr. Western blotting of conditioned medium from HEK293T cells demonstrated that mature protein was secreted for each of these variants (Figure 4C). Therefore, we carried out large-scale production of each variant and used IMAC to purify the N-terminally tagged AMH (cleaved pro-mature complexes). In a SMAD1/5/9-responsive assay in HEK293T cells, AMH_SCUT_ dose-dependently stimulated transcriptional reporter activity, with an EC_50_ 3 ng/mL (Figure 4D). Several of the single-point mutants (Gln^484^Met, Gly^533^Ser, and Leu^535^Thr) induced small increases in AMH potency and/or efficacy (Figure 4D). These effects, however, were more pronounced when compound mutants were tested. Indeed, the Gln^484^Met/Gly^533^Ser double mutant increased AMH potency six-fold, while the Gln^484^Met/Leu^535^Thr double mutant showed 70% greater efficacy than the AMH_SCUT_ control (Figure 4E). To further characterize the double mutants, we assessed their activity in COV434 cells transfected with a SMAD1/5/9-responsive transcriptional reporter and AMHR2. In this assay, AMH_SCUT_ potency (EC_50_ 4 ng/mL) was enhanced 5- to 10-fold by incorporating the Gln^484^Met/Leu^535^Thr (EC_50_ 0.8 ng/mL) or Gln^484^Met/Gly^533^Ser (EC_50_ 0.4 ng/mL) double mutations, respectively (Figure 4F). Furthermore, the Gln^484^Met/Leu^535^Thr double mutant displayed enhanced efficacy, relative to the AMH_SCUT_ control (Figure 4F).

### 3.4 | Mutation of the putative type I receptor-binding site identifies AMH variants with enhanced activity

**FIGURE 5:**
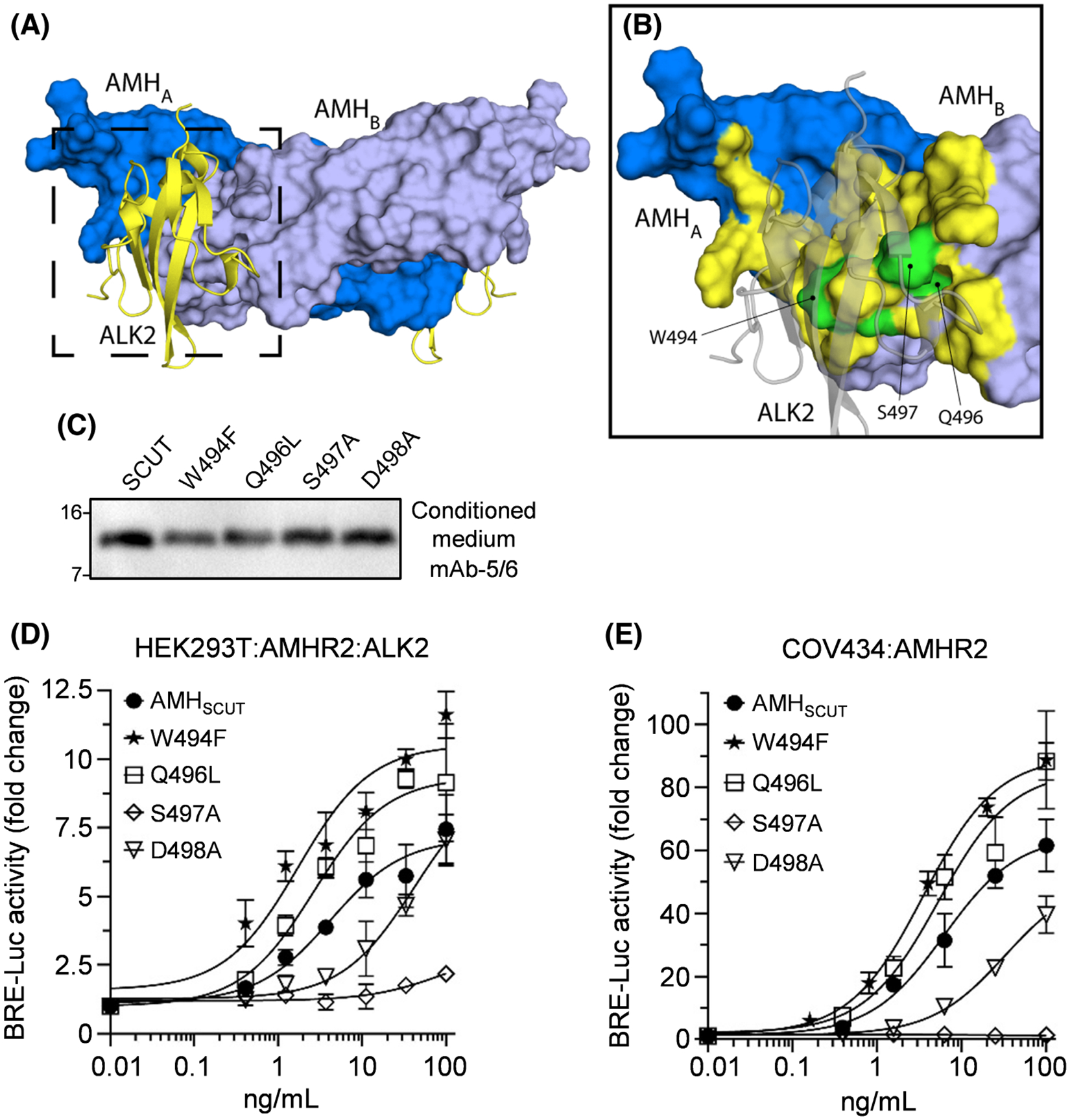
Modifications to the type I site. (A) Structure of dimeric human mature AMH (PDB ID 7L0J) bound at each wrist epitope by a model of the ALK2 extracellular domain. Each monomer of the AMH homodimer is colored in a different shade of blue. Each ALK2 is colored yellow. (B) Close-up view of the putative ALK2-binding site on one side of the AMH homodimer. Atoms of AMH within 5 Å of ALK2 are colored yellow, while some of the key residues targeted by mutagenesis are colored green. (C) Residues in the putative type I receptor-binding epitope of AMH were mutated in the AMH_SCUT_ construct using *in vitro* mutagenesis. Conditioned medium from HEK293T cells transfected with AMH_SCUT_ or type I mutant constructs was analyzed by Western blotting using mAb-5/6, targeted to the AMH mature domain, with samples run under reducing conditions. (D, E) Dose–response curves of SMAD1/5/9-responsive luciferase reporter (BRE-Luc) activity following treatment of cells with IMAC purified AMH_SCUT_ or type I mutants. (D) HEK293T cells transfected with BRE-Luc, AMHR2, and ALK2. (E) COV434 cells transfected with BRE-Luc and AMHR2. Luciferase activity is presented as the mean ± SD of triplicates from representative experiments, relative to an adjusted value of 1.0 for the mean of the control wells. Experiments were repeated >3 times. AMH, anti-Müllerian hormone; AMHR2, AMH type II receptor; IMAC, immobilized metal affinity chromatography.

Focusing on the fingertips and wrist (pre-helix/helix) regions of AMH (Figure 5A), we next performed a mutagenesis study to identify domains/residues involved in type I receptor binding and to determine whether these domains/residues could be modified to generate gain-of-function variants. Interestingly, AMH shares type I receptor specificity with other TGF-β proteins, including BMP2 and BMP6.^49^ However, these BMPs are much more potent activators of the SMAD1/5/9 transcription pathway, suggesting that they bind ALK2/ALK3 with higher affinity than AMH. Thus, we substituted fingertip and wrist regions between these BMPs and AMH and assessed the effects on activity.

The first variant generated substituted the AMH fingertip 1/2 loop (^473^AERS^476^) with that of BMP2 (^307^DVGWNDW^313^) (Figure S3), as the WXXW motif present in all TGF-β superfamily ligands, except AMH, is important for type I receptor binding.^23,50^ Although the AMH_BMP2 chimera_ was secreted by HEK293T cells (Figure S4), it had limited activity in COV434 cells transfected with a SMAD1/5/9-responsive transcriptional reporter and AMHR2 (Figure S4). Similar results were obtained when we substituted sections of the AMH pre-helix loop (^493^GWPQSD^498^ or ^499^RNPRY^503^) with the corresponding regions from BMP6 (^446^SFPLNA^451^ or ^452^HMNAT^456^) (Figure S3). The resulting variants, termed AMH_BMP6 chimera-1_ and AMH_BMP6 chimera-2_, respectively, were secreted by HEK293T cells (note: the higher molecular weight of AMH_BMP6 chimera-2_ is likely due to the inclusion of an N-glycosylation site present within this portion of BMP6^49^) (Figure S4). However, in the COV434 reporter assay, AMH_BMP6 chimera-1_ had reduced activity, relative to AMH_SCUT_, and AMH_BMP6 chimera-2_ was inactive (Figure S4).

As large domain swaps reduced or abrogated AMH activity, we switched approach to introducing single BMP2/BMP6 residues across the AMH wrist domain (Figure S3). AMH_SCUT_ variants, Trp^494^Phe, Gln^496^Leu, Ser^497^Ala, or Asp^498^Ala, were secreted at wild-type levels (Figure 5C) and, following purification by IMAC, their activity was assessed in HEK293T cells transfected with a SMAD1/5/9-responsive transcriptional reporter, together with AMHR2 and ALK2 (Figure 5D). AMH_SCUT_ activity (EC_50_ 4 ng/mL) was reduced five-fold by the incorporation of the Asp^498^Ala mutation and almost completely abrogated by the Ser^497^Ala substitution. In contrast, AMH_SCUT_ activity was substantially enhanced by incorporation of the Gln^496^Leu (EC_50_ 2 ng/mL) or Trp^494^Phe (EC_50_ 1 ng/mL) substitutions, respectively (Figure 5D). The Gln^496^Leu and Trp^494^Phe variants also displayed enhanced efficacy, relative to the AMH_SCUT_ control (Figure 5D). Together, these findings represent the first characterization of AMH residues involved in type I receptor binding and demonstrate that gain-of-function can be achieved by the incorporation of single BMP2/BMP6 residues into the AMH pre-helix.

As AMH can also signal via ALK3,^51–53^ we tested the same variants in HEK293T cells transfected with a SMAD1/5/9-responsive transcriptional reporter, together with AMHR2 and ALK3 (Figure S5); and also in COV434 cells transfected with a SMAD1/5/9-responsive transcriptional reporter and AMHR2, but without over-expressing any type I receptors (Figure 5E). The activity of each variant relative to the AMH_SCUT_ control was comparable in both assays to what was observed in the HEK293T assay using ALK2 over-expression (Figure 5D), confirming that the differences in activity are not restricted to a condition of ALK2 over-expression.

### 3.5 | Effect of combining type I and type II mutants with enhanced activity

**FIGURE 6:**
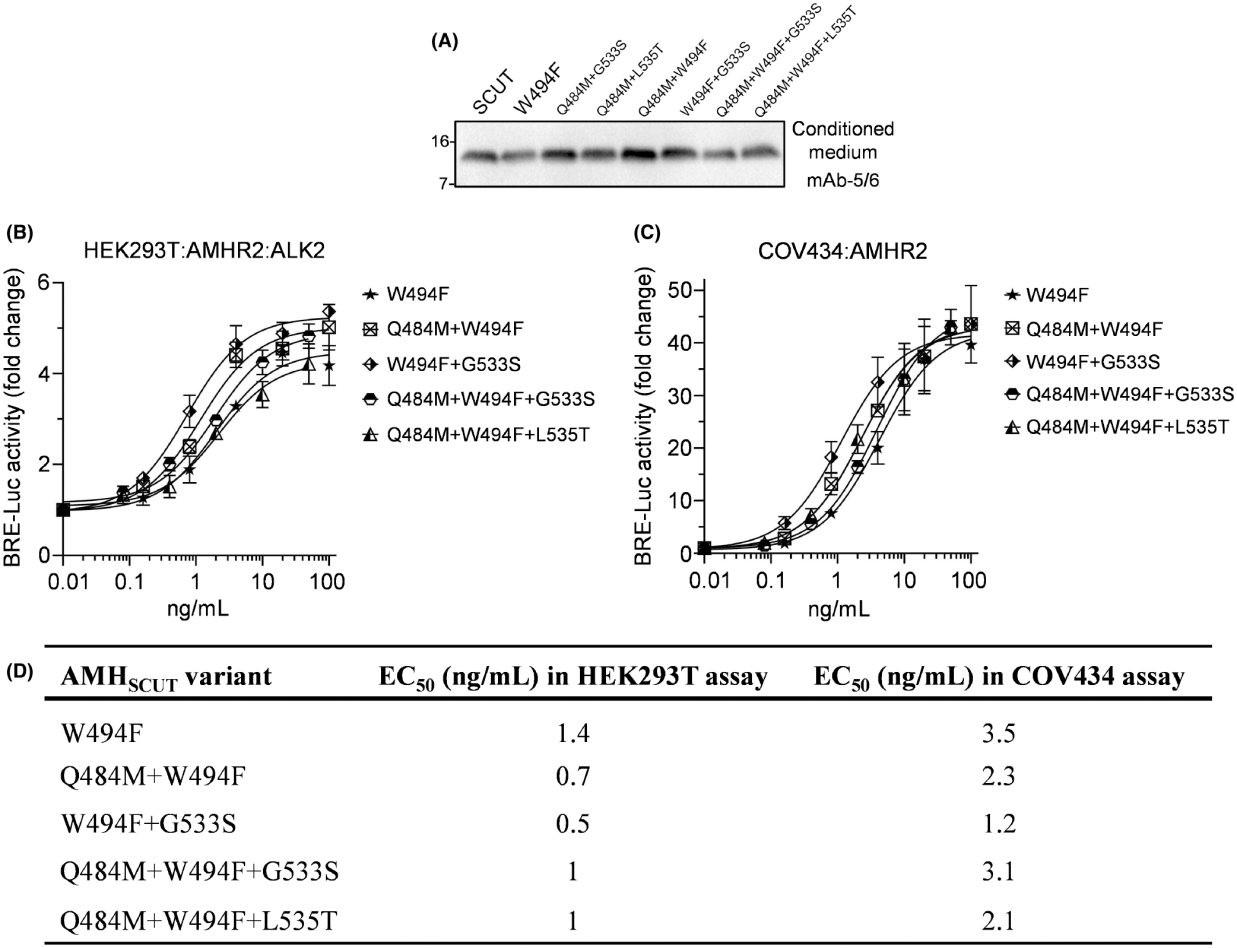
Combined type I and type II activating mutations. (A) Residues in the AMHR2-binding and putative type I receptor-binding epitopes of AMH were mutated in the AMH_SCUT_ construct using *in vitro* mutagenesis. Conditioned medium from HEK293T cells transfected with AMH_SCUT_ or receptor-binding mutant constructs was analyzed by Western blotting using mAb-5/6, targeted to the AMH mature domain, with samples run under reducing conditions. (B, C) Dose–response curves of SMAD1/5/9-responsive luciferase reporter (BRE-Luc) activity following treatment of cells with IMAC purified AMH_SCUT_ or receptor-binding mutants. (B) HEK293T cells transfected with BRE-Luc, AMHR2, and ALK2. (C) COV434 cells transfected with BRE-Luc and AMHR2. Luciferase activity is presented as the mean ± SD of triplicates from representative experiments, relative to an adjusted value of 1.0 for the mean of the control wells. Experiments were repeated >3 times. (D) EC_50_ values of combined type I and type II activating mutations in luciferase reporter assays from (B, C). AMH, anti-Müllerian hormone; AMHR2, AMH type II receptor; IMAC, immobilized metal affinity chromatography.

Having identified gain-of-function mutations at both the type I (pre-helix) and type II (knuckle) receptor interfaces, we next assessed whether combining type I and type II mutations would lead to additive or synergistic increases in AMH activity. Using the AMH Trp^494^Phe variant (Figure 5) as our starting point, we generated: Gln^484^Met/Trp^494^Phe, Trp^494^Phe/Gly^533^Ser, Gln^484^Met/Trp^494^Phe/Gly^533^Ser and Gln^484^Met/Trp^494^Phe/Leu^535^Thr variants. Each of these variants was transiently expressed in HEK293T cells (Figure 6A), and following purification by IMAC, activity was tested in HEK293T cells transfected with a SMAD1/5/9-responsive transcriptional reporter, together with AMHR2 and ALK2 (Figure 6B). In this assay, the Trp^494^Phe variant stimulated transcriptional reporter activity with an EC_50_ of 1.4 ng/mL. Surprisingly, combining mutations that enhanced type I and type II receptor binding only resulted in additive, rather than synergistic, effects on potency. Notably, the Gln^484^Met/Trp^494^Phe variant had an EC_50_ of 0.7 ng/mL, while the Trp^494^Phe/Gly^533^Ser variant had an EC_50_ of 0.5 ng/mL (Figure 6B,D). When treating COV434 cells transfected with a SMAD1/5/9-responsive transcriptional reporter and AMHR2 (Figure 6C), the Trp^494^Phe variant stimulated transcriptional reporter activity with an EC_50_ of 3.5 ng/mL, whereas the Gln^484^Met/Trp^494^Phe variant had an EC_50_ of 2.3 ng/mL, and the Trp^494^Phe/Gly^533^Ser variant had an EC_50_ of 1.2 ng/mL (Figure 6C,D). These results indicate that signaling complex stability can be improved by modulating the interaction between AMH and either the type I receptor or AMHR2, suggesting a step-wise assembly mechanism in which enhancement in the affinity of the ligand for either receptor can increase AMH potency beyond the natural level.

### 3.6 | Computational analysis of changes in AMH activity following modification of the type I and type II receptor-binding sites

**FIGURE 7:**
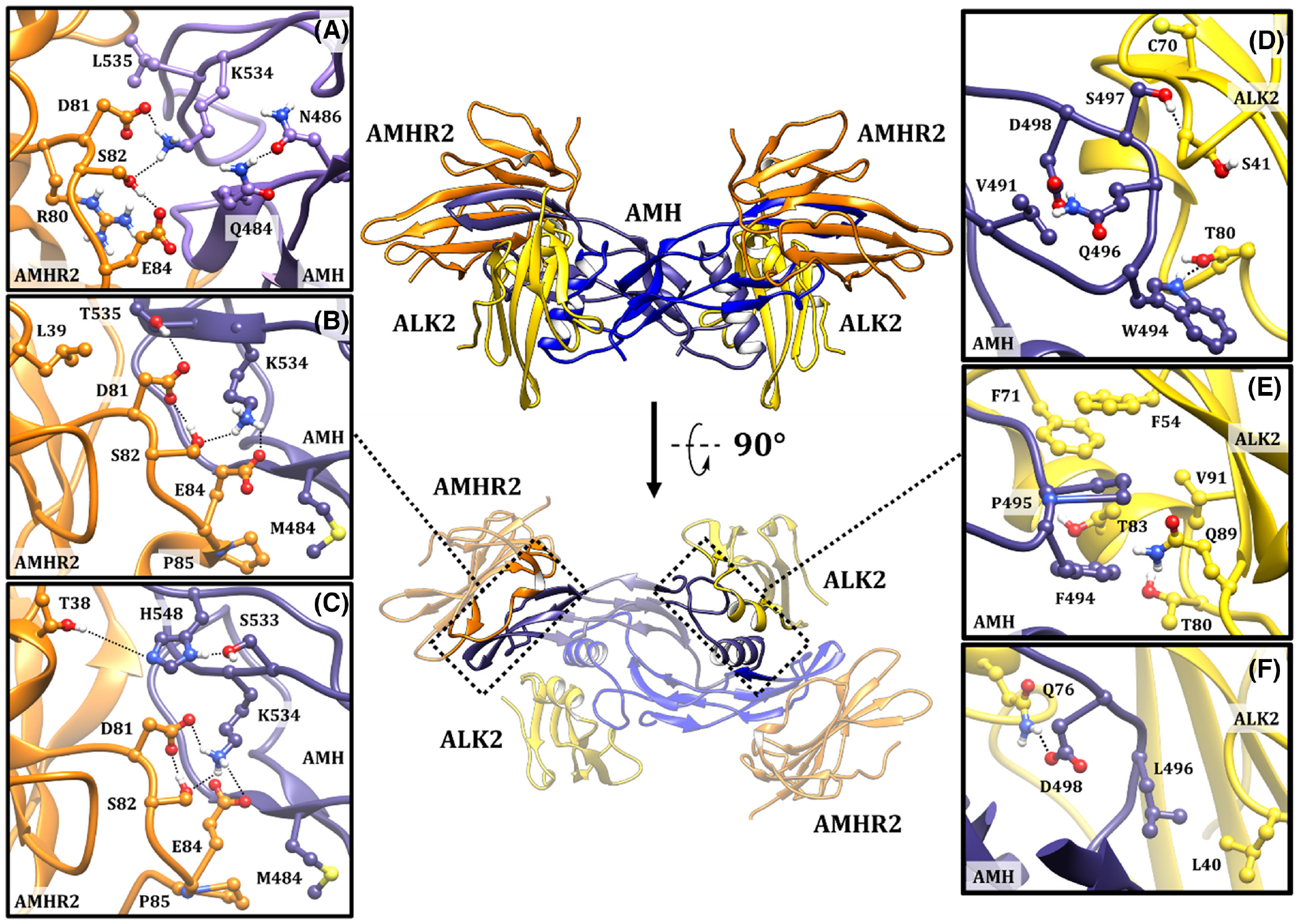
Modeled structural basis for enhanced activity of AMH mutants. Representative structures from MD simulations with enlarged surface residues at the type II site in (A) wild-type, (B) Gln^484^Met/Leu^535^Thr, and (C) Gln^484^Met/Gly^533^Ser AMH variants. Representative structures from MD simulations with enlarged surface residues at the type I site in (D) wild-type, (E) Trp^494^Phe, and (F) Gln^496^Leu AMH variants. Each monomer of the AMH homodimer is colored in a different shade of blue. AMHR2 is colored orange. ALK2 is colored yellow. Specific residues involved in ligand-receptor interactions are shown in ball-and-stick representation and labeled. Dotted black lines between chains indicate hydrogen bonds. AMH, anti-Müllerian hormone; AMHR2, AMH type II receptor; MD, molecular dynamics.

To identify changes in the structure of the ligand-receptor complex resulting from the mutations of the AMH ligand which demonstrated increased activity, we analyzed the following MD simulations: wild-type AMH, Gln^484^Met/Leu^535^Thr-AMH and Gln^484^Met/Gly^533^Ser-AMH, each bound to AMHR2. We also analyzed wild-type AMH, Trp^494^Phe-AMH, Gln^496^Leu-AMH, and Ser^497^Ala-AMH, each modeled with ALK2 (Figure 7).

Calculated binding free energies revealed that modifications at the type II site which enhanced AMH activity were accompanied by an increase in the ligand-receptor complex stability, particularly after introducing the combined Gln^484^Met/Gly^533^Ser mutations (Table S2). Gln^484^ is located on the very edge of the AMHR2 interface, with its side chain oriented away from the receptor, allowing the formation of an intra-ligand hydrogen bond with Asn^486^ on finger 2 (Figure 7A). Substitution of a polar glutamine at residue 484 with a hydrophobic methionine enables the establishment of van der Waals interactions with Pro^85^ of AMHR2 (Figure 7B), reflected in the increased contribution to the total binding energy by both amino acids; −2.41 kcal mol^−1^ for Met^484^ and −0.94 kcal mol^−1^ for Pro^85^, when compared to wild-type (Table S2). With respect to the Leu^535^Thr mutation, Leu^535^ is positioned in a hydrophobic pocket of AMHR2 unfavorable to polar residues. A switch from leucine to threonine represents a good compromise between retaining the hydrophobic character, through the methyl moiety, and introducing a new functional group with the potential to form hydrogen bonds. Via interacting with Leu^39^ and Asp^81^ of AMHR2 (Figure 7B), AMH residue Thr^535^, relative to the native leucine, increases the residue’s individual energy contribution by −0.98 kcal mol^−1^, but also leads to a stronger ligand-receptor interaction overall (Table S2). Mutation of Gly^533^ to serine does not appear to generate a novel interaction with AMHR2 via Ser^533^, but could rather stabilize the extended beta sheet of finger 3, promoting contacts with the receptor such as that between AMH residue His^548^ and AMHR2 residue Thr^38^, while locking AMH residue Lys^534^ into a favorable orientation for interacting with AMHR2 (Figure 7C).

Looking more closely at the environment of Lys^534^, previously identified as a critical amino acid for AMHR2-binding,^23^ we observed that the introduction of our gain-of-function mutations positively affected the propensity of Lys^534^ to form a salt bridge with Asp^81^ and Glu^84^ and a hydrogen bond with Ser^82^. Although Glu^84^ appears to be favorably oriented towards Lys^534^ in wild-type AMH, during MD simulations it instead created an intra-receptor salt bridge with Arg^80^, causing a negative energetic contribution to ligand binding (+0.22 kcal mol^−1^); whereas Lys^534^ interacted with Asp^81^ on a continual basis, and occasionally with Ser^82^ (Figure 7A; Figure S6). In the Gln^484^Met/Leu^535^Thr mutant, Lys^534^ showed more frequent interactions with Glu^84^ and Ser^82^, by 15% and 20%, respectively (Figure 7B; Figure S6). The most prominent effect was with the Gln^484^Met/Gly^533^Ser mutant, where Lys^534^ formed a permanent salt bridge with both Asp^81^ and Glu^84^ (Figure 7C; Figure S6), making Lys^534^ the dominant AMH residue in the receptor-binding interface (Table S2). Overall, the enhanced activity observed for both the Gln^484^Met/Leu^535^Thr and Gln^484^Met/Gly^533^Ser AMH mutants appears to result from stronger interactions of the individually mutated amino acids, as well as from a stabilizing effect on the electrostatic contacts of Lys^534^ with AMHR2.

Based on the calculated MM-GBSA energy, the interaction of the free wild-type ligand with ALK2 is less favorable than that with AMHR2, consistent with our previously stated assumption that AMH binds its receptors via a non-concerted mechanism (Table S3). The Trp^494^Phe mutation was beneficial to overall AMH potency and efficacy; although the replacement of tryptophan with phenylalanine decreased the individual energy contribution of that residue (Table S3) due to the loss of the hydrogen bond between the tryptophan’s indole and Thr^80^ (Figure 7D; Figure S7). This loss is partially compensated by the CH∙∙∙π interactions with the alkyl side chains of Val^91^, Thr^80^, and Thr^83^; as well as by the hydrogen bond between the backbone C=O of Phe^494^ and side chain of Gln^89^ (Figure S7). The positive effect of the Trp^494^Phe mutation on ligand-receptor stability is primarily manifested in a reduction of polarity in the binding pocket, promoting hydrophobic interactions between AMH residue Pro^495^ and AMHR2 residues Phe^54^ and Phe^71^ (Table S3 and Figure 7E). In wild-type AMH, the side chain of Gln^496^ is oriented towards the interior where it forms intra-ligand hydrogen bonds with Asp^498^ and Val^491^ (Figure 7D; Figure S7). Substitution with the less polar leucine not only enables the establishment of interactions with hydrophobic amino acids of the receptor, such as Leu^40^, but also releases the side chain of Asp^498^ to interact with Gln^76^ (Figure 7F; Figure S7). By forming several hydrogen bonds with receptor amino acids, such as Ser^41^ and Cys^70^ (Figure 7D; Figure S7), Ser^497^ acts as an important capping residue for the interaction of AMH and ALK2. In contrast, the Ser^497^Ala mutant showed loss-of-function, with the calculated MM-GBSA energy showing residue 497 shift from being one of the most dominant amino acids to one that disfavors ligand-receptor interactions, and ultimately resulted in a decrease in the affinity of AMH for ALK2 (Table S3). In summary, the computational analysis provides mechanistic insights into the effects of the experimentally identified point mutations at the type I receptor-binding site.

### 3.7 | Effect of the Ala^515^Val polymorphism on AMH biosynthesis and activity

Ala^515^ is present at the end of the wrist helix of AMH and may play a role in type I receptor binding.^54^ We were interested in this residue because a stable polymorphism (Ala^515^Val) occurs at this site in ~1% of the population.^55^ Western blotting of conditioned medium demonstrated that the Ala^515^Val polymorphism reduced AMH_SCUT_ secretion by 70%, despite equal amounts of precursor being present in the cell lysate (Figure S8). To assess the impact of the Ala^515^Val polymorphism on activity, we carried out IMAC purification and treated HEK293T cells that had been transfected with a SMAD1/5/9-responsive transcriptional reporter, along with AMHR2 and ALK2 (Figure S8). Interestingly, the Ala^515^Val variant was more potent (EC_50_ 2 ng/mL) than the AMH_SCUT_ control (EC_50_ 3 ng/mL) and displayed 25% greater efficacy. A similar result was observed in COV434 cells transfected with a SMAD1/5/9-responsive transcriptional reporter and AMHR2 (Figure S8). The enhanced activity of AMH Ala^515^Val may offset its reduced secretion and explain why this polymorphism is maintained at high levels in the population.

## 4 | DISCUSSION

AMH is a member of the TGF-β superfamily and controls reproductive organ differentiation and ovarian follicular development. Intriguingly, several features of AMH distinguish it from other TGF-β family members. In this study, we characterized the molecular interactions that underlie the synthesis, secretion, and receptor activation of AMH, and used this information to generate potent AMH analogs.

AMH, like other TGF-β family proteins, is synthesized as a large precursor consisting of a signal peptide, an N-terminal prodomain, and a C-terminal mature domain.^56^ AMH has the largest prodomain within the family (57 kDa), primarily due to an ~100-amino acid N-terminal extension. In this study, we assessed the importance of this extended N-terminus by deleting 40 (Pro^30^-Pro^69^) or 90 (Pro^30^-Gly^119^) residues after the signal peptide. Although AMH synthesis was not affected by the deletions, secretion was severely compromised, indicating that this region of the prodomain is important for the correct folding and dimerization of mature AMH. Based on the crystal structures of pro-TGF-β1, pro-myostatin, pro-activin A, and pro-BMP9,^45,57–59^ the region of the AMH prodomain following the N-terminal extension likely consists of two α-helices separated by a linking region. In the existing pro-complex structures,^45,57–59^ the prodomain α2-helix is always involved in hydrophobic interactions with the mature growth factor, while the prodomain α1-helix is critical for folding in a subset of family members (TGF-β1, myostatin and activin A). Alanine scanning mutagenesis of select residues within the putative α1 (Leu^130^-Pro^142^) and α2 (Ala^162^-Gly^169^) helices of the AMH prodomain highlighted the importance of these regions in the correct folding and secretion of the mature growth factor. The importance of the N-terminal portion of the prodomain, including the putative α1 and α2 helices, to AMH biology is further illuminated by the identification by other groups of numerous point mutations through these regions in cases of PMDS, including: Leu^70^Pro, Gly^101^Arg, and Tyr^167^Cys, among others.^3^ Similarly, the PCOS-specific *AMH* variant Pro^151^Ser results in defective protein biosynthesis and retention in the endoplasmic reticulum, leading to reduced AMH secretion.^60^ While our study has identified prodomain regions/residues that mediate AMH synthesis, further structural characterization is required to determine whether pro-AMH adopts a ring-shaped “closed-arm” conformation like pro-TGF-β1,^45^ a V-shaped “open-arm” conformation like pro-myostatin, pro-activin A and pro-BMP9,^57–59^ or a completely novel conformation.

The AMH precursor is cleaved intracellularly by members of the proprotein convertase subtilisin kexin family (PCSK1-PCSK9)^17^ and AMH is secreted from cells non-covalently associated with its prodomain (complex referred to variously as c-AMH or AMH_N,C_^18,61,62^). Interestingly, *in vitro* processing of the human AMH precursor at the proprotein convertase consensus sequence R^448^-A^449^-Q^450^-R^451^ is extremely poor, with only ~10% of total AMH secreted in a cleaved form.^16,17^ We have observed similar results when expressing mouse, cat, and dog AMH *in vitro* (unpublished). The development of specific ELISAs for different AMH forms suggests that processing of this growth factor may be more efficient *in vivo*,^62^ however, it is still not ideal. Inefficient processing may be a means of controlling extracellular matrix interactions, determining receptor binding, or establishing a morphogenic gradient.^56,63,64^ Previously, several groups have improved AMH cleavage significantly by the introduction of a Gln^450^Arg mutation at the cleavage site.^17,46^ In this study, we enhanced processing to 95% by incorporating a theoretically ideal proprotein convertase recognition sequence “ISSRKKRSVSS” (termed SCUT).

The prodomain is displaced from AMH upon bivalent binding to AMHR2.^61^ Recently, Hart *et al*. solved the structure of AMH bound to AMHR2.^23^ The structure showed that AMH adopts the typical “butterfly” architecture of other TGF-β ligands, best envisaged by joining the palms of left and right hands with fingers extending in opposite directions. AMHR2 receptors bind to the “knuckle” region of each AMH monomer, in a similar manner to how activin and BMP ligands bind their type II receptors.^23^ However, differences in AMH (particularly centered around Lys^534^) and AMHR2 are responsible for a highly specific interaction. In previous studies,^65,66^ we used amino acid differences across mammalian species to generate potent analogs of the TGF-β ligands, GDF9 and BMP15. Although the AMHR2-binding epitope of AMH is typically highly conserved, some species (e.g., koala and chicken) harbor distinct residues through this region. Substituting these residues individually into human AMH generated variants (e.g., Gln^484^Met, Gly^533^Ser, and Leu^535^Thr) with slightly enhanced activity. Based on the AMH/AMHR2 structure, these mutations may stabilize the interaction of AMH with AMHR2 in different ways. Computational analysis revealed that the Gln^484^Met mutation replaced hydrogen bonds within the ligand with hydrophobic interactions with AMHR2 residues at the interface, while Leu^535^Thr or Gly^533^Ser substitutions introduced functional groups that can participate in the formation of hydrogen bonds directly, or by promoting other favorable contacts strengthen the binding of AMH to AMHR2. Interestingly, when these mutations were combined (Gln^484^Met/Gly^533^Ser, Gln^484^Met/Leu^535^Thr), human AMH activity was increased 10- and 5-fold, respectively. Thus, introducing residues from other species was sufficient to generate the first gain-of-function human AMH analogs. Whether koala AMH, itself, is more potent than human AMH, requires further consideration. The residues in koala AMHR2 expected to contact these AMH residues are identical to those in human AMHR2, though our koala AMH/AMHR2 model (not shown) suggests the receptor is up to 2 Å closer to the koala ligand than the human ligand.

Based on affinity, it is assumed that AMH first binds AMHR2 and then recruits a BMP type I receptor (either ALK2, ALK3, or ALK6).^51,67–69^ For BMPs, type I receptor binding occurs through extensive contacts in the concave cleft formed by the wrist of one monomer and fingers of the second monomer.^44,50^ However, AMH lacks the two tryptophan residues in the fingertip 1/2 loop that are central to BMP-ALK2/3/6 interactions, implying that AMH may use a different mode of type I receptor binding. In this study, we demonstrated that residues in the pre-helix region of the wrist (Trp^494^, Gln^496^, Ser^497^, and Asp^498^) are particularly important for AMH activity and, therefore, likely mediate type I receptor interactions. Specifically, substituting single BMP2/BMP6 residues into the AMH pre-helix was sufficient to generate gain- (e.g., Trp^494^Phe) or loss-of-function (e.g., Ser^497^Ala) variants. Our modeling suggests that Ser^497^, by participating in the hydrogen bond network, acts as an important capping residue for the AMH-ALK2 interaction. The Gln^496^Leu mutation leads to a change in the orientation of the side chain, allowing for a more efficient interaction with amino acids on the surface of the type I receptor. Finally, mutation of Trp^494^, located in a hydrophobic environment, to a less polar phenylalanine decreases steric clashing and allows for a tighter interaction with ALK2.

Interestingly, combining type I (e.g., Trp^494^Phe) and type II (e.g., Gly^533^Ser) activating mutations did not result in marked additive, or synergistic, increases in AMH potency, suggesting that AMH engages its receptors in a step-wise, non-cooperative manner. Another consideration is that the signaling assays performed in the current study utilized non-covalently associated pro-mature complexes of AMH, as opposed to free mature AMH. The AMH prodomain attenuates AMHR2-binding of the mature domain^61^ and subsequent SMAD phosphorylation.^18^ As the prodomains of TGF-β ligands typically interact with the same mature domain regions as receptors,^50^ the mutations we introduced into the AMH receptor-binding sites may have also altered this association, leading to greater potency.

In summary, we have: (1) demonstrated that the N-terminal extension and α1- (Leu^130^-Pro^142^) and α2- (Ala^162^-Gly^169^) helices of the prodomain are critical for correct folding and secretion of AMH, (2) further enhanced the processing of AMH, (3) generated gain-of-function human AMH variants, based on species differences across the AMHR2-binding site, and (4) identified the putative type I receptor binding site of AMH. Collectively, our results have shown that as few as four amino acid alterations across both the cleavage site and receptor interfaces can significantly increase the production and activity of human AMH. We envisage that these novel AMH analogs will prove useful in translating the biological actions of AMH into clinical applications.^70^

## Supplementary Material

sTable3

sTable2

STable1

sFig7

sFig6

sFig5

sFig8

sFig3

sFig4

sFig2

sFig1

## Data Availability

The data that support the findings of this study are available upon reasonable request to the corresponding author.

## Abbreviations:

ALK: activin receptor-like kinase
AMH: anti-Müllerian hormone
AMHR2: AMH type II receptor
BMP: bone morphogenetic protein
GAPDH: glyceraldehyde-3-phosphate dehydrogenase
IMAC: immobilized metal affinity chromatography
SCUT: super-cut motif
SMAD: *Sma*-and *Mad*-related proteins
MD: molecular dynamics
MM-GBSA: molecular mechanics generalized Born surface area
PCOS: polycystic ovary syndrome
PMDS: persistentMüllerian duct syndrome
TGF-β: transforminggrowth factor-β
