# Supplementary material for: Characterization of the molecular mechanisms that govern anti-Müllerian hormone synthesis and activity
Source: FASEB J. Author manuscript; Available in PMC 2024 Mar 11. (PMC10926428; doi:10.1096/fj.202301335RR)
Supplement: sTable3 [file NIHMS1972931-supplement-sTable3.docx]

**Table S3.** MM-GBSA calculated binding free energies for the AMH-ALK2 complex (Δ*G*_BIND_) from the last 30 ns of trajectories with wild-type, Trp^494^Phe, Gln^496^Leu, and Ser^497^Ala variants. The binding energy decomposition on a *per-residue* basis for each individual amino acid is given in kcal mol^–1^. The data include amino acid residues that promote binding the most (separated by a dotted line), as well as those important for the discussion. The introduced mutations are shaded in different colours.

| **Modifications to the type I site** | | | | | | | | | | | | | | | |
| --- | --- | --- | --- | --- | --- | --- | --- | --- | --- | --- | --- | --- | --- | --- | --- |
| Wild-type | | | | Trp^494^Phe | | | | Gln^496^Leu | | | | Ser^497^Ala | | | |
| Δ*G*_BIND_ = −63.62 kcal mol^−1^ | | | | Δ*G*_BIND_ = −80.89 kcal mol^−1^ | | | | Δ*G*_BIND_ = −84.73 kcal mol^−1^ | | | | Δ*G*_BIND_ = −62.37 kcal mol^−1^ | | | |
| AMH | | ALK2 | | AMH | | ALK2 | | AMH | | ALK2 | | AMH | | ALK2 | |
| Pro495 | −4.80 | Met79 | −3.97 | Pro495 | −6.10 | Phe71 | −5.38 | Ser497 | −4.87 | Pro84 | −7.43 | Trp494 | −4.70 | Gln89 | −4.75 |
| **Ser497** | −4.59 | Glu49 | −3.54 | Arg543 | −5.57 | Leu40 | −4.75 | Trp494 | −4.70 | Met79 | −7.16 | Arg516 | −4.18 | Pro87 | −4.48 |
| Arg499 | −4.03 | Phe54 | −3.18 | Ser497 | −5.03 | Ser41 | −4.37 | Pro495 | −4.41 | Gln89 | −5.66 | Pro495 | −3.46 | Pro84 | −3.26 |
| Arg475 | −4.02 | Ser41 | −2.99 | Arg499 | −3.97 | Glu49 | −4.15 | **Leu496** | −4.18 | Ser41 | −3.35 | Pro501 | −2.86 | Met79 | −3.23 |
| **Trp494** | −3.57 | Thr83 | −2.87 | Ser538 | −3.43 | Gln72 | −3.86 | Tyr503 | −3.66 | Leu40 | −3.28 | Pro461 | −2.78 | Ser86 | −2.88 |
| Tyr503 | −2.02 | Gln89 | −2.83 | **Phe494** | −2.66 | Val73 | −3.38 | Pro461 | −2.77 | Thr83 | −3.17 | His547 | −2.64 | Leu40 | −2.83 |
| **Gln496** | −1.81 | Gln76 | −2.80 | Glu541 | −2.53 | Phe54 | −3.18 | Arg516 | −2.72 | Gln76 | −3.14 | Arg475 | −2.45 | Lys82 | −2.28 |
| Ala473 | −1.80 | Thr80 | −2.09 | Gln496 | −2.19 | Cys70 | −2.59 | Val508 | −2.47 | Phe54 | −3.02 | Glu474 | −2.36 | Gln76 | −1.87 |
| Val515 | −1.57 | Pro84 | −2.00 | Pro461 | −2.05 | Pro84 | −2.49 | Arg475 | −1.89 | Thr80 | −2.33 | Tyr503 | −2.27 | Phe54 | −1.78 |
| Arg516 | −1.57 | Cys70 | −1.71 | Ser476 | −1.77 | Met79 | −1.71 | Leu509 | −1.37 | Lys82 | −2.16 | Val515 | −2.00 | Gln72 | −1.69 |
|  |  | Phe71 | −1.66 |  |  |  |  |  |  |  |  | **Ala497** | 0.13 |  |  |
|  |  | Leu40 | −1.06 |  |  |  |  |  |  |  |  |  |  |  |  |
