# Supplementary material for: Characterization of the molecular mechanisms that govern anti-Müllerian hormone synthesis and activity
Source: FASEB J. Author manuscript; Available in PMC 2024 Mar 11. (PMC10926428; doi:10.1096/fj.202301335RR)
Supplement: sTable2 [file NIHMS1972931-supplement-sTable2.docx]

**Table S2.** MM-GBSA calculated binding free energies for the AMH-AMHR2 complex (Δ*G*_BIND_) from the last 30 ns of trajectories with wild-type, Gln^484^Met/Leu^535^Thr, and Gln^484^Met/Gly^533^Ser variants. The binding energy decomposition on a *per-residue* basis for each individual amino acid is given in kcal mol^–1^. The data include amino acid residues that promote binding the most (separated by a dotted line), as well as those important for the discussion. The introduced mutations are shaded in different colours, while the contribution of Lys^534^ is represented in bold red.

| **Modifications to the type II site** | | | | | | | | | | | |
| --- | --- | --- | --- | --- | --- | --- | --- | --- | --- | --- | --- |
| Wild-type | | | | Gln^484^Met/Leu^535^Thr | | | | Gln^484^Met/Gly^533^Ser | | | |
| Δ*G*_BIND_ = −72.06 kcal mol^−1^ | | | | Δ*G*_BIND_ = −76.58 kcal mol^−1^ | | | | Δ*G*_BIND_ = −89.50 kcal mol^−1^ | | | |
| AMH | | AMHR2 | | AMH | | AMHR2 | | AMH | | AMHR2 | |
| Arg543 | −6.86 | Arg33 | −8.69 | Ile544 | −6.22 | Arg33 | −6.45 | **Lys534** | −7.40 | Arg33 | −7.89 |
| Glu481 | −4.70 | Arg80 | −5.21 | Ile479 | −4.57 | Pro100 | −3.70 | Ile544 | −5.82 | Asp81 | −6.44 |
| Ile544 | −3.99 | Glu75 | −3.62 | **Lys534** | −3.51 | Val32 | −3.05 | Ile479 | −4.15 | Arg97 | −5.02 |
| Ile479 | −3.48 | Pro30 | −2.92 | Leu478 | −3.25 | Ser101 | −3.04 | Leu535 | −4.10 | Glu84 | −3.19 |
| Leu478 | −3.44 | Val32 | −2.87 | Ile537 | −3.21 | Ile64 | −2.72 | Thr482 | −3.68 | Ile64 | −2.88 |
| **Lys534** | −2.22 | Ile64 | −2.06 | Ala546 | −3.07 | Arg80 | −2.03 | Ser545 | −3.62 | Val32 | −2.62 |
| Ile537 | −2.22 | Phe62 | −1.60 | Glu481 | −2.90 | Phe62 | −1.89 | Glu474 | −3.36 | Phe62 | −2.12 |
| Ala546 | −2.02 | Met76 | −1.38 | Leu539 | −2.71 | Met76 | −1.77 | **Met484** | −3.35 | Pro85 | −1.79 |
| Glu542 | −1.84 | Leu106 | −1.26 | Tyr483 | −2.63 | Ser82 | −1.67 | Tyr483 | −3.20 | Met76 | −1.79 |
| Leu539 | −1.66 | Ser82 | −1.25 | **Thr535** | −2.48 | Pro85 | −1.62 | Leu478 | −3.13 | Leu106 | −1.72 |
| **Leu535** | −1.50 | Asp81 | −1.02 | **Met484** | −2.47 | Asp81 | −0.96 | **Ser533** | −0.26 | Ser82 | −0.01 |
| **Gln484** | −0.06 | Pro85 | −0.68 |  |  | Glu84 | −0.04 |  |  |  |  |
| **Gly533** | 0.18 | Glu84 | 0.22 |  |  |  |  |  |  |  |  |
