# Supplementary material for: Characterization of the molecular mechanisms that govern anti-Müllerian hormone synthesis and activity
Source: FASEB J. Author manuscript; Available in PMC 2024 Mar 11. (PMC10926428; doi:10.1096/fj.202301335RR)
Supplement: STable1 [file NIHMS1972931-supplement-STable1.docx]

**Table S1. Human AMH primers**

| **Primer** | **Sequence (5’-3’)** |
| --- | --- |
| hAMH_5’_NotI | CTAGGCGGCCGCATGCGGGACCTGCCTCTCACCAG |
| hAMH_3’_HindIII | CTAGAAGCTTTCACCGGCAGCCACACTCGGTG |
| hAMH_SCUT_S | GAGAAAGAAACGCTCAGTCTCATCAAGCGCGGGGGCCACCGCCGCCGACGGGCCG |
| hAMH_SCUT_AS | GCGTTTCTTTCTCGATGAGATACCCGGCCCGCGCGGATCCCGCCCGCGCCAC |
| ΔPro^30^-Pro^69^_S | CTGCGGGTGGTGGGGGCT |
| ΔPro^30^-Gly^119^_S | GCCTGGCTGCAGGACCCT |
| ΔPro^30^_AS | ATGATGATGATGATGATGGAGC |
| hAMH_L133A_S | CAGCGCCTGGTGGTCGCACACCTGGAGGAAGTG |
| hAMH_L133A_AS | CACTTCCTCCAGGTGTGCGACCACCAGGCGCTG |
| hAMH_H134A_S | GCGCCTGGTGGTCCTAGCCCTGGAGGAAGTGACC |
| hAMH_H134A_AS | GGTCACTTCCTCCAGGGCTAGGACCACCAGGCGC |
| hAMH_L135A_S | CCTGGTGGTCCTACACGCGGAGGAAGTGACCTGGG |
| hAMH_L135A_AS | CCCAGGTCACTTCCTCCGCGTGTAGGACCACCAGG |
| hAMH_E137A_S | GGTCCTACACCTGGAGGCAGTGACCTGGGAGCC |
| hAMH_E137A_AS | GGCTCCCAGGTCACTGCCTCCAGGTGTAGGACC |
| hAMH_V138A_S | CTACACCTGGAGGAAGCGACCTGGGAGCCAAC |
| hAMH_V138A_AS | GTGTTGGCTCCCAGGTCGCTTCCTCCAGGTGTAGG |
| hAMH_W140A_S | CTGGAGGAAGTGACCGCGGAGCCAACACCCTCGC |
| hAMH_W140A_AS | CGAGGGTGTTGGCTCCGCGGTCACTTCCTCCAGG |
| hAMH_L164A_S | CCAGAGCTGGCGCTGGCGGTGCTGTACCCTGGG |
| hAMH_L164A_AS | CCAGGGTACAGCACCGCCAGCGCCAGCTCTGGGGGGC |
| hAMH_Y167A_S | GGCGCTGCTGGTGCTGGCCCCTGGGCCTGGCCC |
| hAMH_Y167A_AS | GGGCCAGGCCCAGGGGCCAGCACCAGCAGCGCC |
| hAMH_G169A_S | CTGGTGCTGTACCCTGCGCCTGGCCCTGAGGTC |
| hAMH_G169A_AS | GACCTCAGGGCCAGGCGCAGGGTACAGCACCAG |
| hAMH_RAQR_S | CCGGGTCGGGCACAACGCAGCGCGGGG |
| hAMH_RAQR_AS | CCCCGCGCTGCGTTGTGCCCGACCCGG |
| hAMH_RKKR_S | CGCGGGCCGGGTCGGAAGAAACGCAGCGCGGGGGCC |
| hAMH_RKKR_AS | GGCCCCCGCGCTGCGTTTCTTCCGACCCGGCCCGCG |
| hAMH_Q484M_S | CATCCCCGAGACCTACATGGCCAACAATTGCCAG |
| hAMH_Q484M_AS | CTGGCAATTGTTGGCCATGTAGGTCTCGGGGATG |
| hAMH_G533D_S | GCCCACCGCCTACGCGGACAAGCTGCTCATCAG |
| hAMH_G533D_AS | CTGATGAGCAGCTTGTCCGCGTAGGCGGTGGGC |
| hAMH_G533S_S | CCGCCTACGCGAGCAAGCTGCTCATCAGCC |
| hAMH_G533S_AS | GATGAGCAGCTTGCTCGCGTAGGCGGTGGGC |
| hAMH_L535T_S | GCCTACGCGGGCAAGACGCTCATCAGCCTGTCGG |
| hAMH_L535T_AS | CCGACAGGCTGATGAGCGTCTTGCCCGCGTAGGC |
| hAMH_W494F_S | GGGCGTGTGCGGCTTTCCTCAGTCCGACCGC |
| hAMH_W494F_AS | GCGGTCGGACTGAGGAAAGCCGCACACGCCCTGGC |
| hAMH_Q496L_S | GTGTGCGGCTGGCCTCTGTCCGACCGCAACCCG |
| hAMH_Q496L_AS | CGGGTTGCGGTCGGACAGAGGCCAGCCGCACAC |
| hAMH_S497A_S | GCGGCTGGCCTCAGGCCGACCGCAACCCGCGC |
| hAMH_S497A_AS | GCGCGGGTTGCGGTCGGCCTGAGGCCAGCCGCACACG |
| hAMH_D498A_S | GGCTGGCCTCAGTCCGCCCGCAACCCGCGCTAC |
| hAMH_D498A_AS | GTAGCGCGGGTTGCGGGCGGACTGAGGCCAGCC |
| AMH_BMP2 chimera_S | GATGTGGGCTGGAATGACTGGGTACTCATCCCCGAGACCTACCAG |
| AMH_BMP2 chimera_AS | CCAGTCATTCCAGCCCACATCGCGGAGGTCTACGCTGAGCTCGCG |
| AMH_BMP6 chimera-1_S | TGCTCCTTCCCACTCAACGCACGCAACCCGCGCTACGGCAAC |
| AMH_BMP6 chimera-1_AS | TGCGTTGAGTGGGAAGGAGCACACGCCCTGGCAATTGTTGGC |
| AMH_BMP6 chimera-2_S | TCCGACCACATGAATGCAACCGGCAACCACGTGGTGCTGCTG |
| AMH_BMP6 chimera-2_AS | GGTTGCATTCATGTGGTCGGACTGAGGCCAGCCGCA |
| hAMH_A515V_S | GCTGAAGATGCAGGTTCGTGGGGCCGCCCTGGC |
| hAMH_A515V_AS | CAGGGCGGCCCCACGAACCTGCATCTTCAGCAGC |
