# Supplementary material for: Characterization of the molecular mechanisms that govern anti-Müllerian hormone synthesis and activity
Source: FASEB J. Author manuscript; Available in PMC 2024 Mar 11. (PMC10926428; doi:10.1096/fj.202301335RR)
Supplement: sFig7 [file NIHMS1972931-supplement-sFig7.docx]

| 1. Wild-type | 1. Trp^494^Phe |
| --- | --- |
| 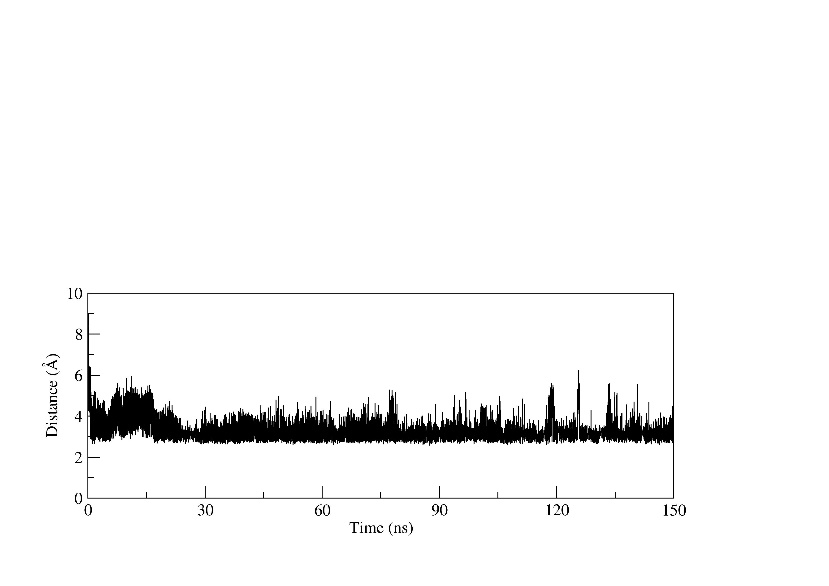 | 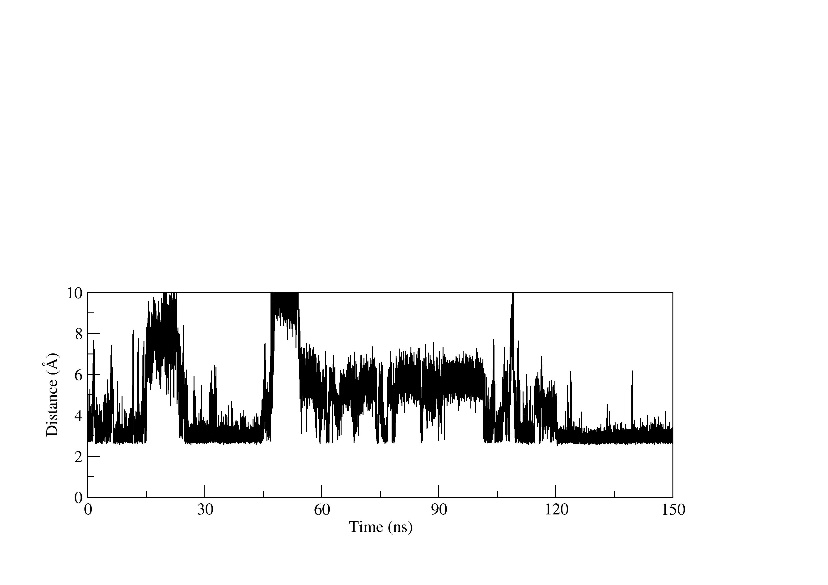 |
| Trp^494^[NH]∙∙∙Thr^80^[OH] | Phe^494^[C=O]∙∙∙Gln^89^[NH_2_] |
| 1. Wild-type | 1. Gln^496^Leu |
| 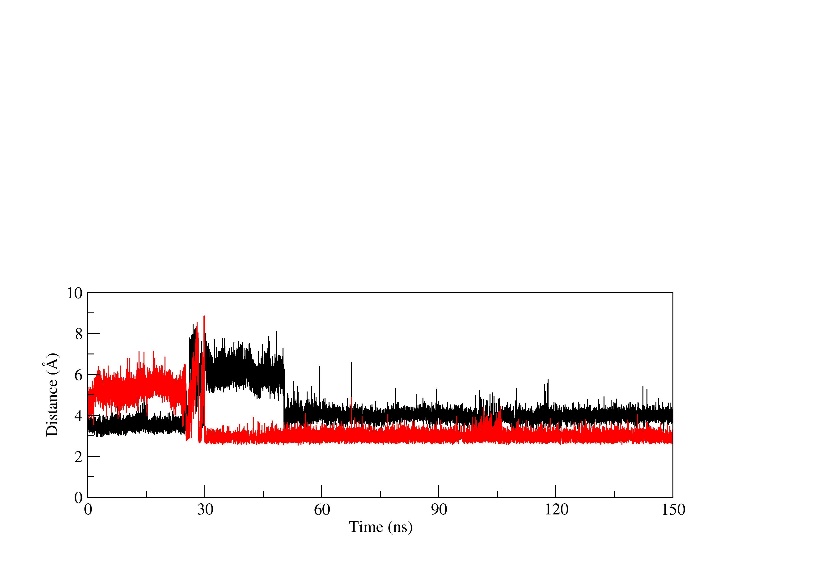 | 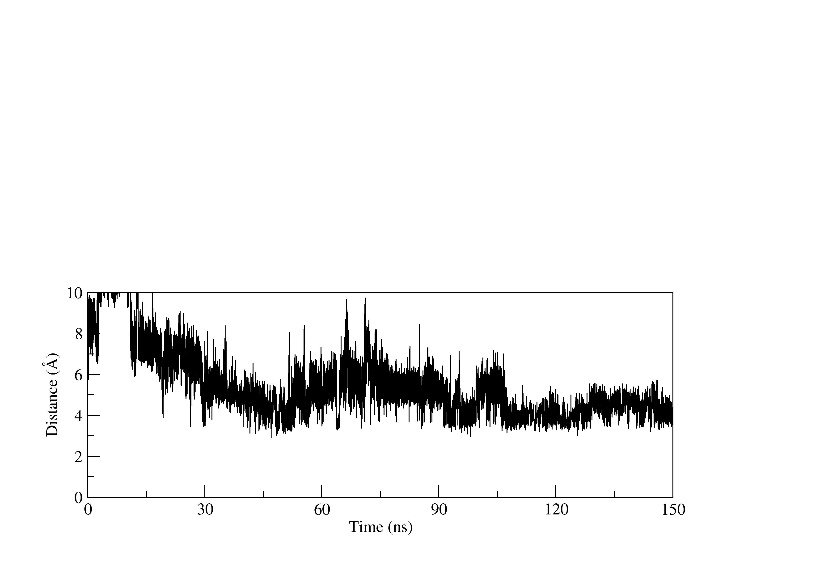 |
| Gln^496^[NH]∙∙∙Asp^498^[COO^−^] in black  Gln^496^[NH]∙∙∙Val^491^[C=O] in red | Asp^498^[COO^−^]∙∙∙Gln^76^[NH] |
| 1. Wild-type |  |
| 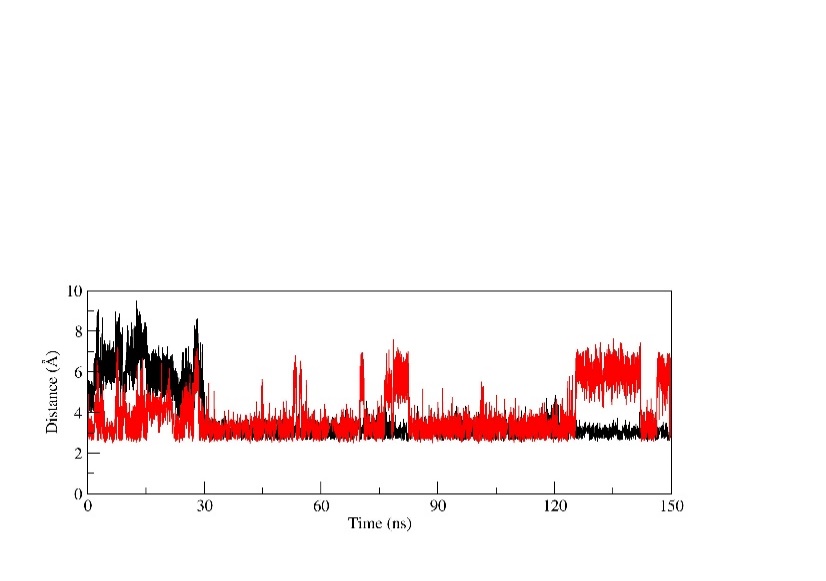 | 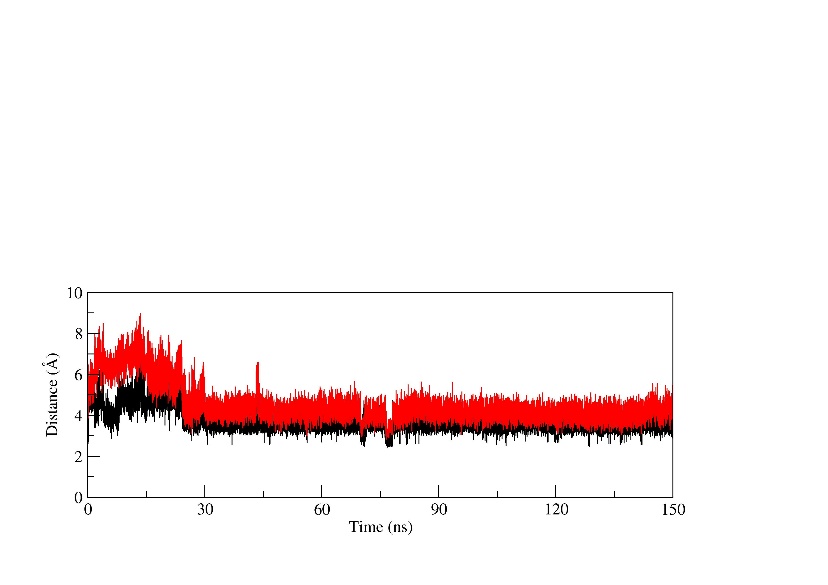 |
| Ser^497^[NH]∙∙∙Ser^41^[C=O] in black  Ser^497^[OH]∙∙∙Ser^41^[OH] in red | Ser^497^[OH]∙∙∙Cys^70^[C=O] in black  Ser^497^[OH]∙∙∙Cys^70^[S] in red |

**Figure S7.** Evolution of distances between various hydrogen bond donors and acceptors during the course of MD simulations with A), C), E) wild-type, B) Trp^494^Phe and D) Gln^496^Leu AMH variants in complex with ALK2 receptor.
