# Supplementary material for: Characterization of the molecular mechanisms that govern anti-Müllerian hormone synthesis and activity
Source: FASEB J. Author manuscript; Available in PMC 2024 Mar 11. (PMC10926428; doi:10.1096/fj.202301335RR)
Supplement: sFig6 [file NIHMS1972931-supplement-sFig6.docx]

| 1. Wild-type |
| --- |
| 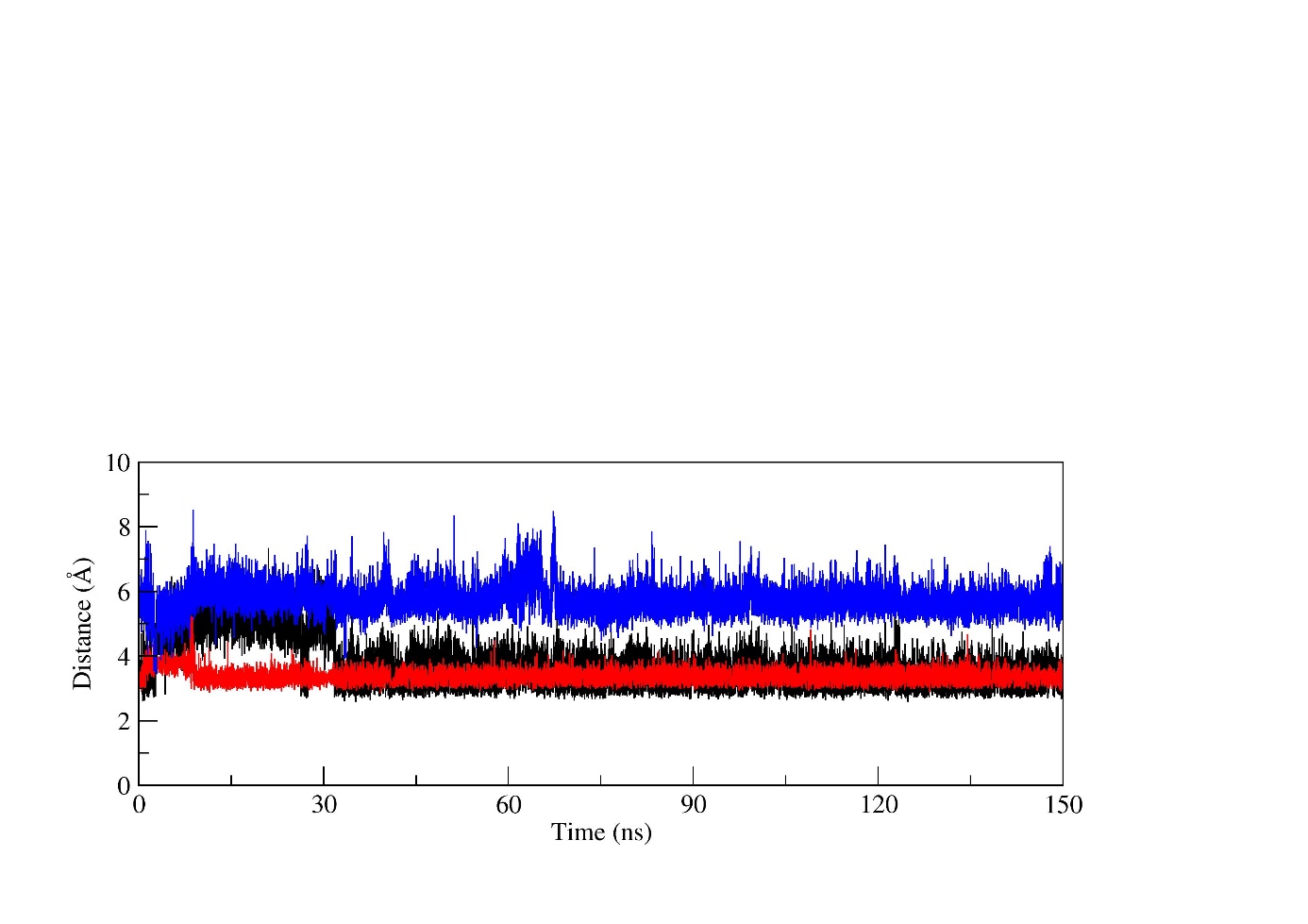 |
| 1. Gln^484^Met/Leu^535^Thr |
| 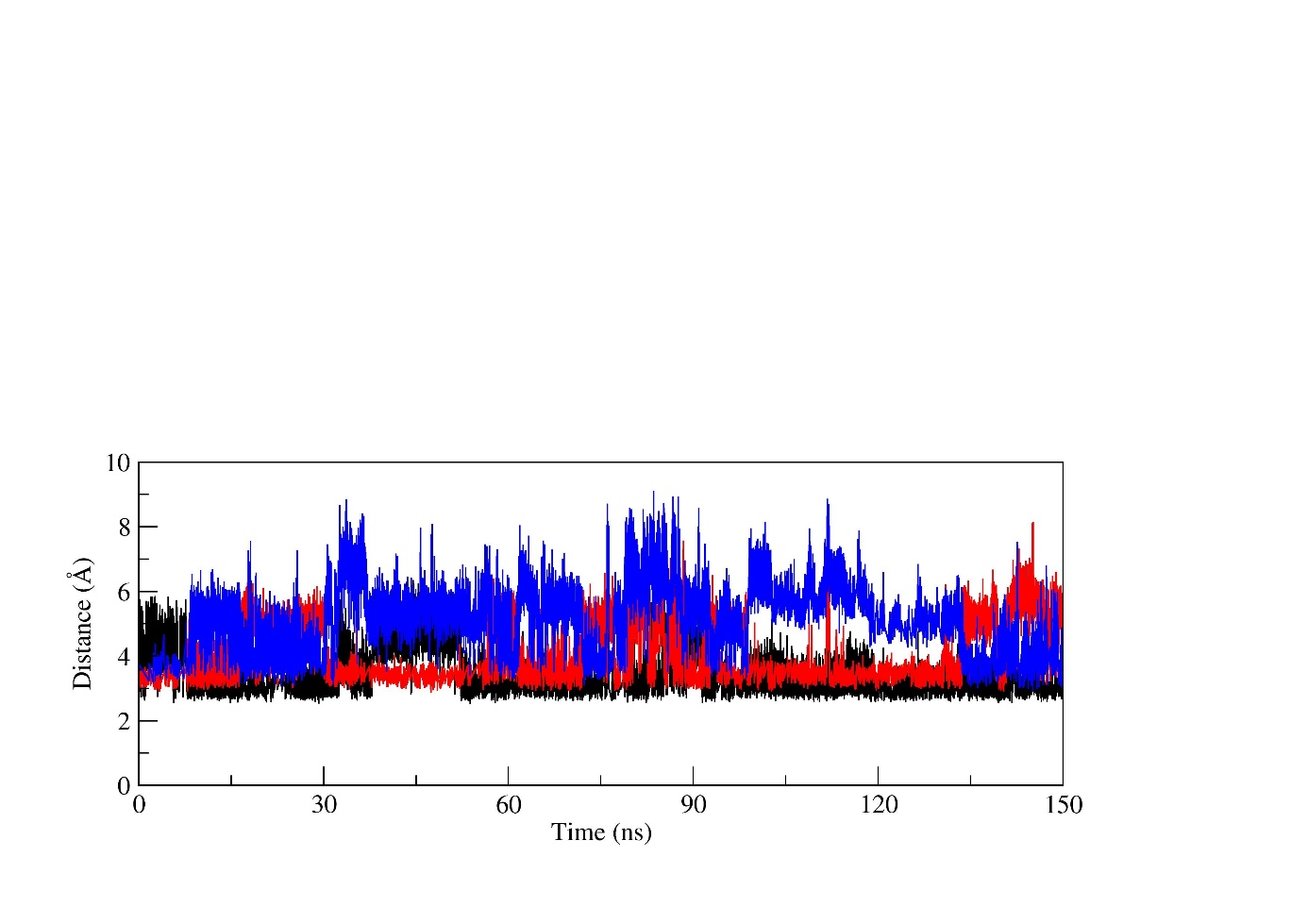 |
| 1. Gln^484^Met/Gly^533^Ser |
| 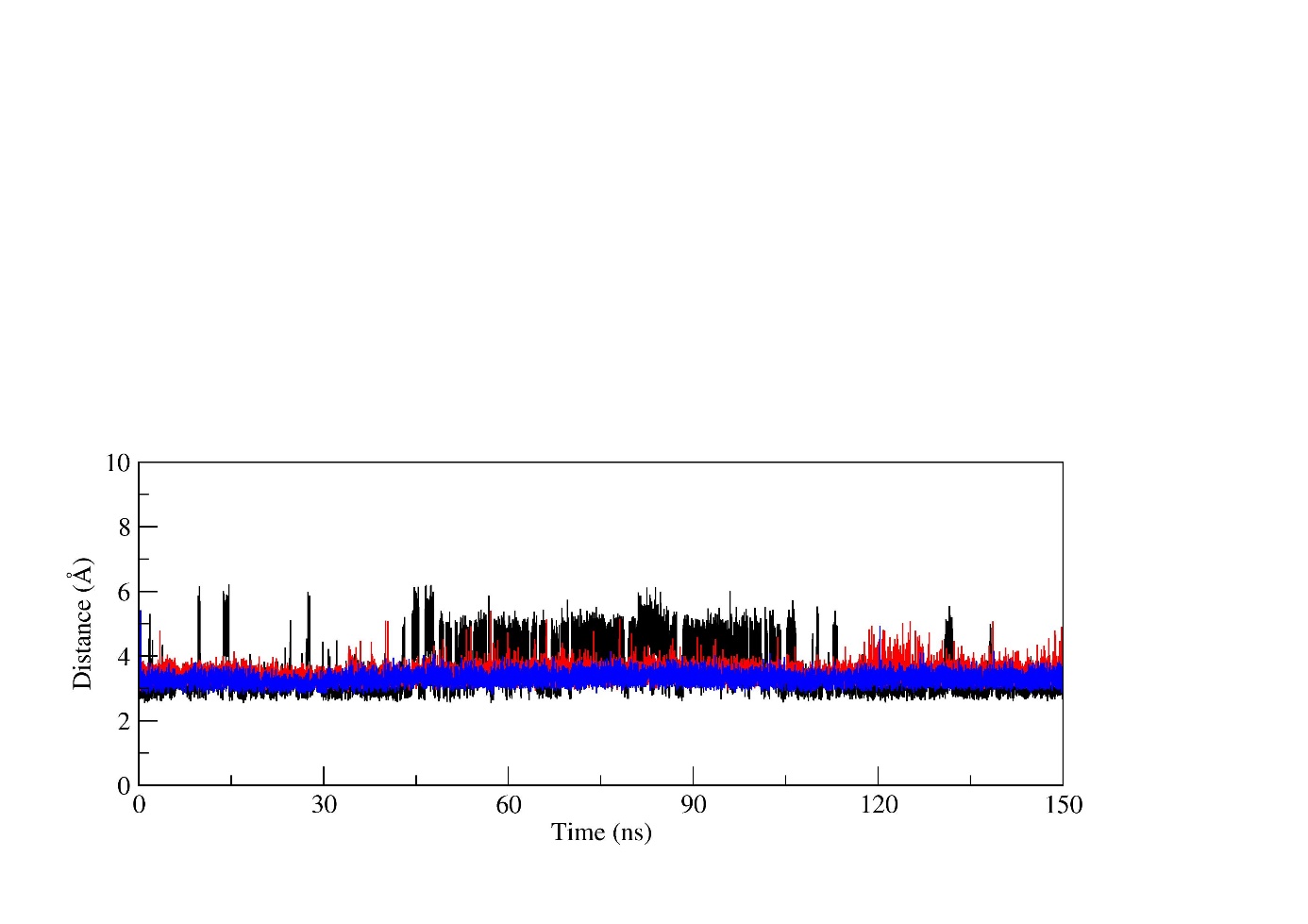 |

**Figure S6.** Evolution of distances between the NH_3_^+^ of Lys^534^ and side chain heteroatoms of Asp^81^ (in red), Ser^82^ (in black), and Glu^84^ (in blue) during the course of MD simulations with A) wild-type, B) Gln^484^Met/Leu^535^Thr, and C) Gln^484^Met/Gly^533^Ser AMH variants in complex with AMHR2 receptor.
