# Supplementary material for: Characterization of the molecular mechanisms that govern anti-Müllerian hormone synthesis and activity
Source: FASEB J. Author manuscript; Available in PMC 2024 Mar 11. (PMC10926428; doi:10.1096/fj.202301335RR)
Supplement: sFig5 [file NIHMS1972931-supplement-sFig5.docx]

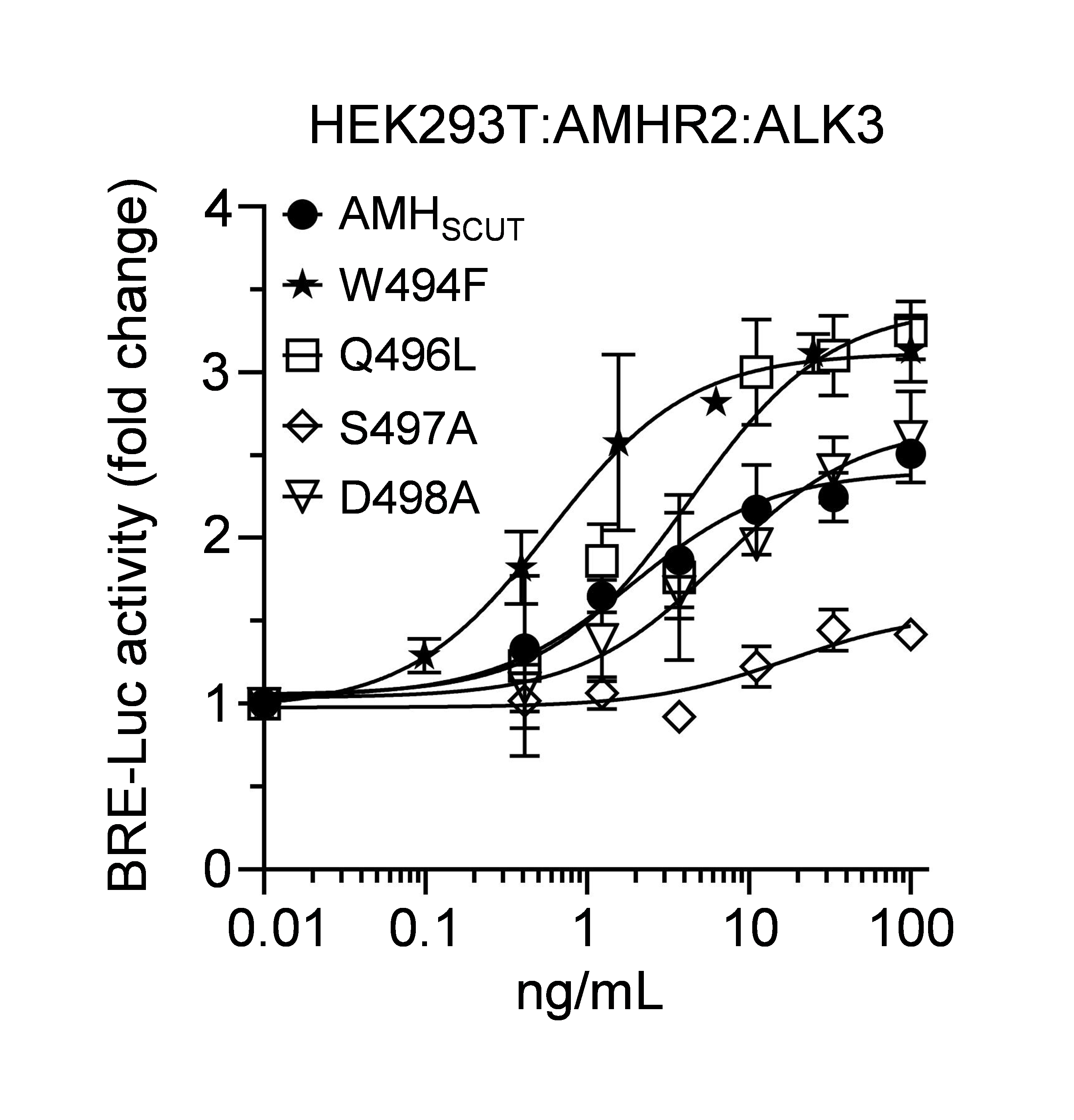


**Figure S5. Activity of type I mutants with ALK3 over-expression.** Dose-response curve of SMAD1/5/9-responsive luciferase reporter (BRE-Luc) activity following treatment of HEK293T cells transfected with BRE-Luc, AMHR2 and ALK3, with IMAC purified AMH_SCUT_ or type I mutants. Luciferase activity is presented as the mean ± S.D. of triplicates from a representative experiment, relative to an adjusted value of 1.0 for the mean of the control wells. The experiment was repeated >3 times.
