# Supplementary material for: Characterization of the molecular mechanisms that govern anti-Müllerian hormone synthesis and activity
Source: FASEB J. Author manuscript; Available in PMC 2024 Mar 11. (PMC10926428; doi:10.1096/fj.202301335RR)
Supplement: sFig8 [file NIHMS1972931-supplement-sFig8.docx]

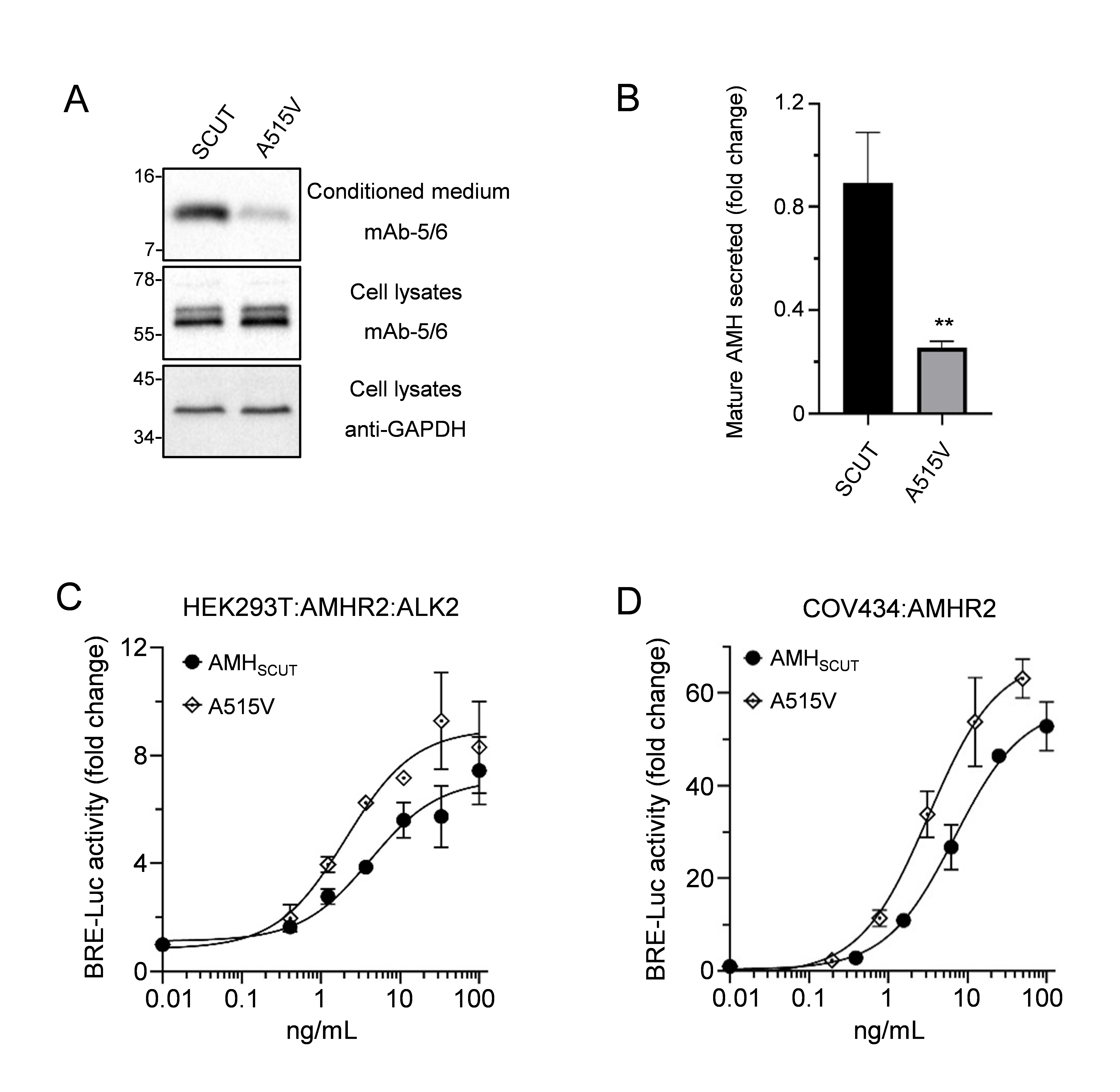


**Figure S8. Effect of the A515V polymorphism.** (A) The A515V polymorphism was substituted into the AMH_SCUT_ construct using *in vitro* mutagenesis. To determine the effects on AMH biosynthesis, conditioned medium and cell lysates from HEK293T cells transfected with either AMH_SCUT_ or A515V constructs were analysed by Western blotting, with samples run under reducing conditions. Conditioned medium samples were probed with mAb-5/6, targeted to the AMH mature domain. Cell lysates were probed with mAb-5/6, or anti-GAPDH as a loading control. (B) Densitometric quantification of AMH_SCUT_ or A515V mature domain secretion was performed using the Bio-Rad ChemiDoc^TM^ MP system and Image Lab^TM^ software (Bio-Rad). Data is presented as the mean ± S.D. of three separate transfections, with representative Western blots shown in (A). Stars indicate a significant difference of P < 0.01 (**) when compared to AMH_SCUT_. (C, D) Dose-response curves of SMAD1/5/9-responsive luciferase reporter (BRE-Luc) activity following treatment of cells with IMAC purified AMH_SCUT_ or the A515V variant. (C) HEK293T cells transfected with BRE-Luc, AMHR2 and ALK2. (D) COV434 cells transfected with BRE-Luc and AMHR2. Luciferase activity is presented as the mean ± S.D. of triplicates from representative experiments, relative to an adjusted value of 1.0 for the mean of the control wells. Experiments were repeated >3 times.
