# Supplementary material for: Characterization of the molecular mechanisms that govern anti-Müllerian hormone synthesis and activity
Source: FASEB J. Author manuscript; Available in PMC 2024 Mar 11. (PMC10926428; doi:10.1096/fj.202301335RR)
Supplement: sFig3 [file NIHMS1972931-supplement-sFig3.docx]

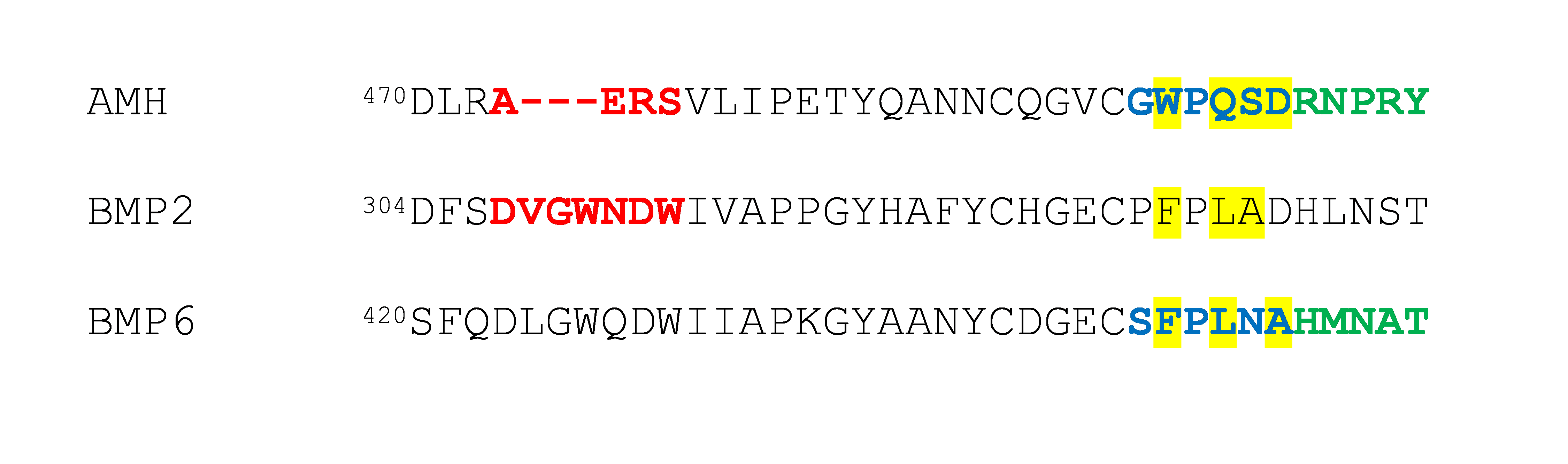


**Figure S3. Sequence alignment of the “wrist” region of human AMH, BMP2 and BMP6.** Sequences were obtained from the UniProt database (www.uniprot.org/) and mature domains were aligned using ClustalW (Conway Institute, University College Dublin, Dublin, Ireland). Different colours indicate which AMH residues were substituted for BMP2 or BMP6 residues in the constructs AMH_BMP2 chimera_ (*red*), AMH_BMP6 chimera-1_ (*blue*) and AMH_BMP6 chimera-2_ (*green*). AMH residues highlighted (*yellow*) were individually substituted for the corresponding residues from BMP2 or BMP6.
