# Supplementary material for: Characterization of the molecular mechanisms that govern anti-Müllerian hormone synthesis and activity
Source: FASEB J. Author manuscript; Available in PMC 2024 Mar 11. (PMC10926428; doi:10.1096/fj.202301335RR)
Supplement: sFig4 [file NIHMS1972931-supplement-sFig4.docx]

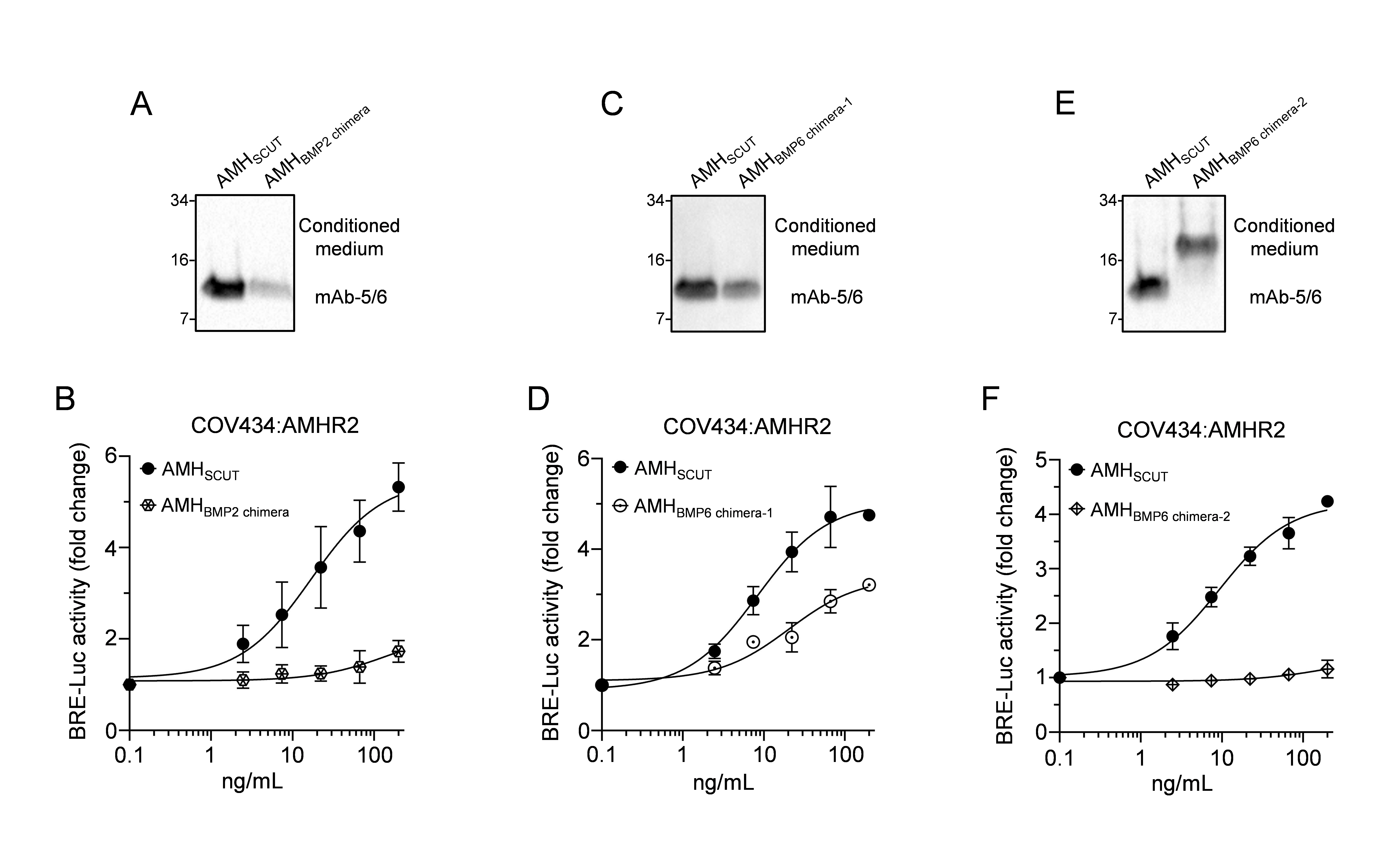


**Figure S4. Putative type I site domain swap chimeras.** (A, C, E) Residues in the putative type I receptor-binding epitope of AMH were mutated in the AMH_SCUT_ construct using *in vitro* mutagenesis. Conditioned medium from HEK293T cells transfected with AMH_SCUT_ or type I mutant constructs was analysed by Western blotting using mAb-5/6, targeted to the AMH mature domain, with samples run under reducing conditions. (B, D, F) Dose-response curves of SMAD1/5/9-responsive luciferase reporter (BRE-Luc) activity following treatment with IMAC purified AMH_SCUT_ or type I mutants, of COV434 cells transfected with BRE-Luc and AMHR2. Luciferase activity is presented as the mean ± S.D. of triplicates from representative experiments, relative to an adjusted value of 1.0 for the mean of the control wells. Experiments were repeated >3 times.
