# Supplementary material for: Characterization of the molecular mechanisms that govern anti-Müllerian hormone synthesis and activity
Source: FASEB J. Author manuscript; Available in PMC 2024 Mar 11. (PMC10926428; doi:10.1096/fj.202301335RR)
Supplement: sFig2 [file NIHMS1972931-supplement-sFig2.docx]

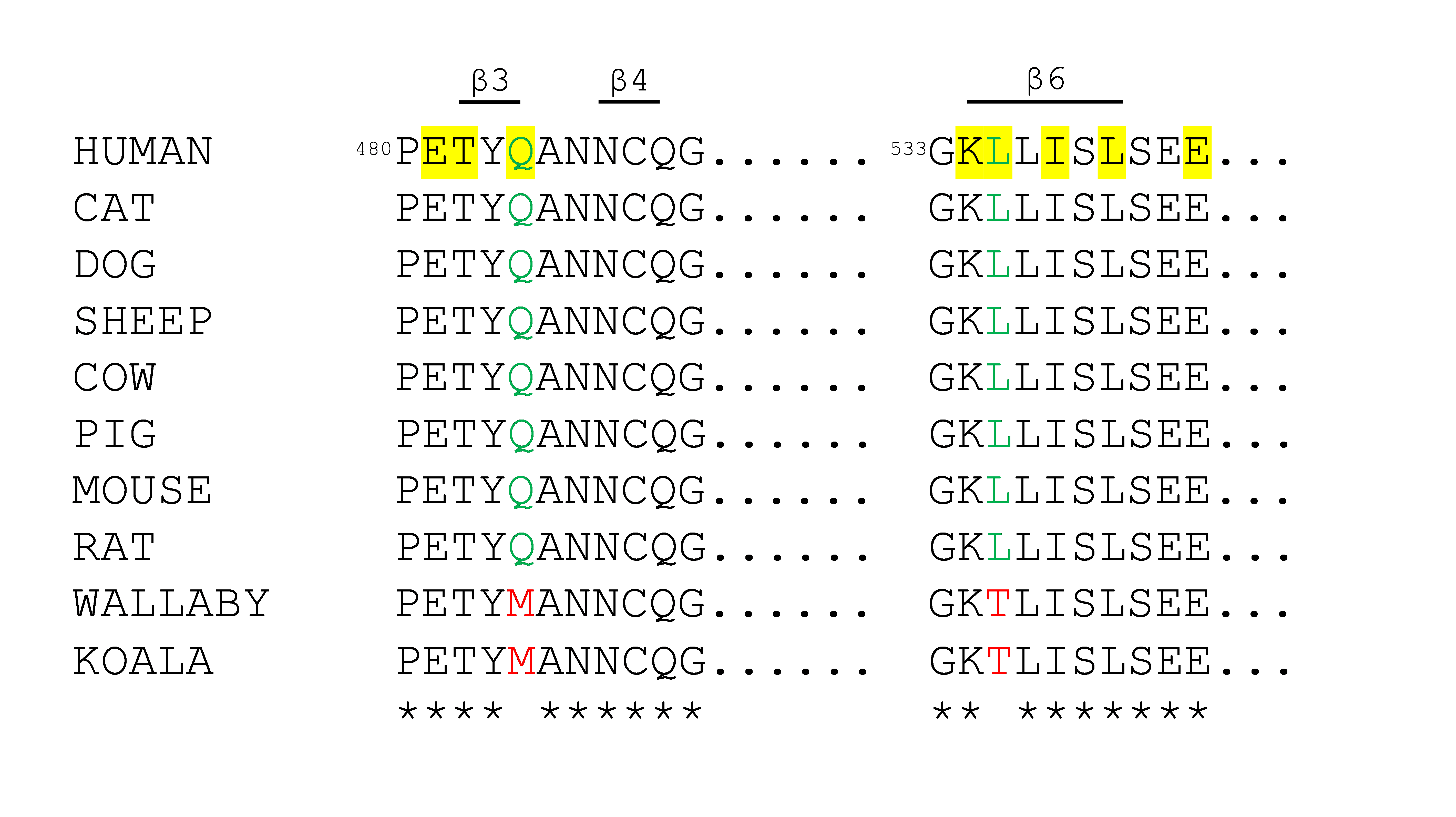


**Figure S2. Sequence alignment of select regions of the AMH mature domain across species.** Sequences were obtained from the UniProt database (www.uniprot.org/) and mature domains were aligned using ClustalW (Conway Institute, University College Dublin, Dublin, Ireland). Human AMH residues that contact AMHR2 ^(23)^ are highlighted (*yellow*). Residues at the AMHR2 interface that differ between mammals and marsupials are indicated (*green/red*).
