# Supplementary material for: Characterization of the molecular mechanisms that govern anti-Müllerian hormone synthesis and activity
Source: FASEB J. Author manuscript; Available in PMC 2024 Mar 11. (PMC10926428; doi:10.1096/fj.202301335RR)
Supplement: sFig1 [file NIHMS1972931-supplement-sFig1.docx]

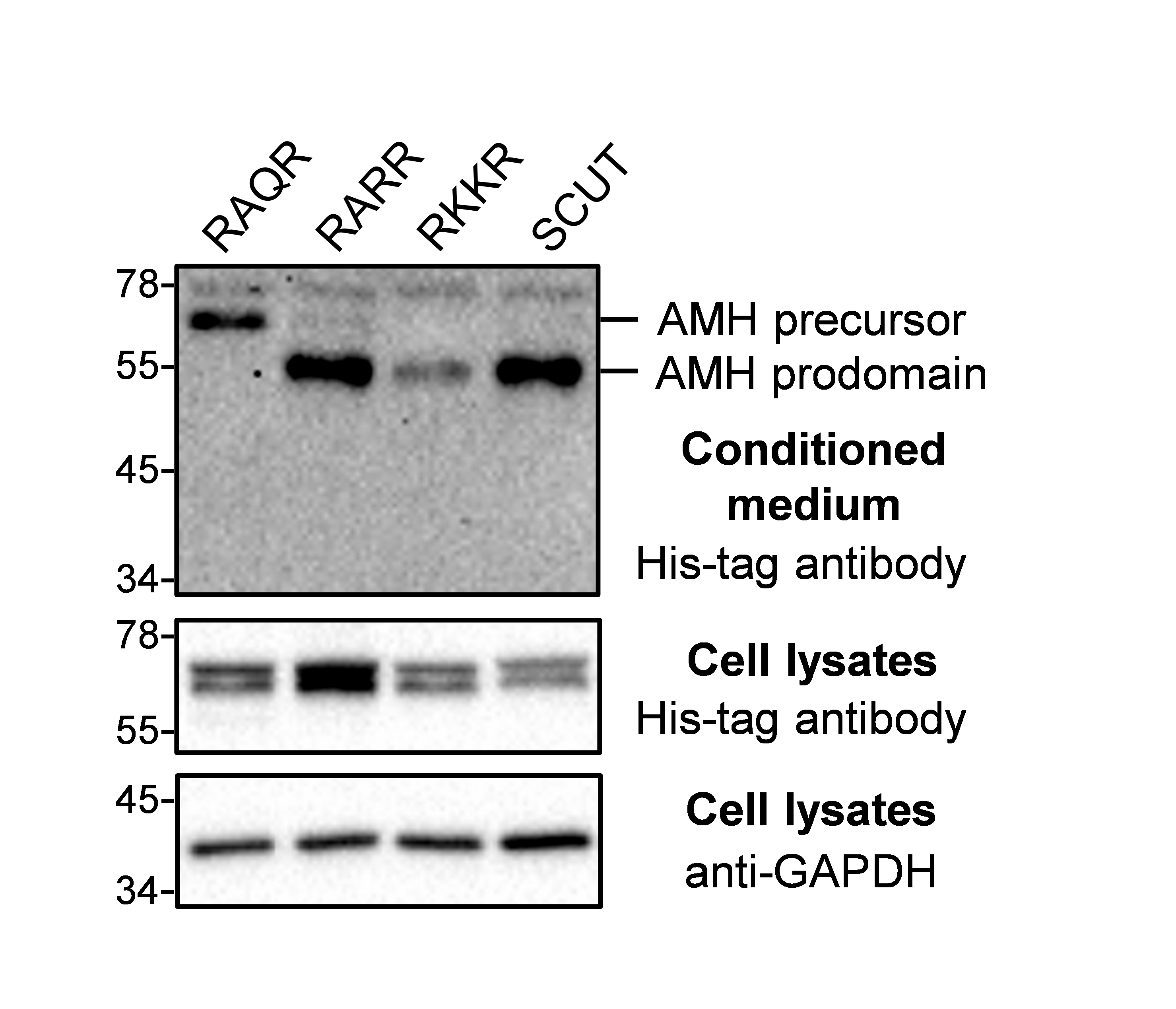


**Figure S1. AMH prodomain secretion with cleavage site variants.** To determine the effect of modifying the AMH cleavage site, conditioned medium and cell lysates from HEK293T cells transfected with either wild-type or mutant constructs were analysed by Western blotting, with samples run under reducing conditions. Conditioned medium samples were probed with a monoclonal His-tag antibody, targeted to a 6×His epitope-tag located at the N-terminus of the AMH prodomain. Cell lysates were probed with the His-tag antibody, or anti-GAPDH as a loading control.
